# Labeled ingredient composition and literature-reported hazard signals in children's toothpastes available through selected UAE retail sources

**DOI:** 10.3389/froh.2026.1932715

**Published:** 2026-08-26

**Authors:** Sally Manla, Manal Al Halabi, Abiola Senok, Rania Nassar, Anas Salami, Iyad Hussein, Mawlood Kowash

**Affiliations:** Dubai Health, Mohammed Bin Rashid University of Medicine and Health Sciences, Dubai, United Arab Emirates

**Keywords:** children's toothpaste, fluoride, natural toothpaste, herbal toothpaste, organic toothpaste, regulatory labeling

## Abstract

**Background:**

Young children frequently swallow toothpaste during brushing, making the composition of ingredients and the context of exposure relevant to pediatric oral care.

**Objective:**

To conduct a descriptive survey of children's toothpastes available through selected UAE retail sources and to develop a structured evidence map of literature-reported biological effects associated with labeled ingredients, without performing product-level safety or risk assessment.

**Methods:**

We surveyed children's toothpastes marketed in the UAE and cataloged labeled ingredients. An evidence-mapping review was conducted to identify published scientific and regulatory reports of biological effects associated with these ingredients. Evidence was classified by source, exposure route, dose relevance, population relevance, evidence consistency, and relevance to routine pediatric toothpaste use.

**Results:**

Sixty-five products were identified, including conventionally marketed toothpastes and those labeled as natural, organic, or herbal (NOHT). Both categories contained diverse ingredients, with substantial overlap in excipients such as surfactants, preservatives, humectants, and flavoring agents. Fluoride labeling was more common among conventional products in this sample (a formulation feature and labeling practice, not a safety comparison). Literature-reported biological effects varied widely by ingredient and were derived from regulatory assessments, human studies, animal models, *in-vitro* experiments, and other exposure contexts, many of which were not directly representative of routine pediatric toothpaste use.

**Conclusions:**

In this UAE market sample, conventional and NOHT children's toothpastes shared multiple ingredients for which biological effects have been reported under varying exposure conditions. These findings represent structured hazard identification and evidence mapping rather than quantitative risk assessment. Product-level safety cannot be determined without ingredient concentration and exposure data, and marketing descriptors such as “natural” or “herbal” should not be interpreted as indicators of safety.

## Introduction

Dental caries remains one of the most prevalent chronic conditions of childhood and is commonly managed through regular toothbrushing with appropriate toothpaste (1). Fluoridated toothpaste is widely recommended because of its established anticaries benefit when used correctly (2–4). At the same time, young children frequently ingest toothpaste during brushing, resulting in repeated low-level oral exposure to fluoride and other formulation ingredients (5, 6).

Children's toothpastes contain multiple functional excipients, including abrasives, surfactants, humectants, preservatives, thickeners, flavoring agents, and colorants. Many of these ingredients have been studied in diverse experimental, clinical, occupational, or regulatory contexts, where biological effects have been reported under specific exposure conditions (6, 7). Interpretation of such findings requires careful attention to the route of exposure, dose, frequency, formulation, and population susceptibility (8, 9).

Consumer interest in products marketed as “natural,” “organic,” or “herbal” has increased, often reflecting perceptions of reduced chemical exposure or increased safety. However, these descriptors are not consistently defined in cosmetic or oral-care labeling frameworks and do not inherently reflect toxicological or exposure-based risk. Natural or plant-derived ingredients may have biological activity, including sensitization potential, and synthetic excipients used in conventional products are often subject to regulatory safety evaluation within defined use conditions (10–12).

Despite widespread toothpaste use, limited region-specific data are available on the composition of children's toothpastes in the United Arab Emirates (UAE). Product availability in the UAE reflects a heterogeneous mix of multinational brands, regional distributors, and e-commerce offerings, which may differ from those in other markets. Against this background, the present study aimed to (i) describe the labeled ingredient composition of children's toothpastes available through selected UAE retail sources and (ii) map literature-reported biological effects associated with those ingredients using a structured, hierarchical evidence-mapping framework, without conducting product-level safety or risk assessment.

## Materials and methods

### Study design

This study combined a cross-sectional market survey of children's toothpastes available in the UAE with a structured evidence-mapping review of published scientific and regulatory literature related to labeled toothpaste ingredients. The study was designed for descriptive hazard identification and contextual interpretation rather than exposure modeling, dose–response analysis, or quantitative risk characterization.

### Market survey of children's toothpastes

Between December 2024 and March 2025, children's toothpastes were identified through in-person retail sampling in Dubai, United Arab Emirates, complemented by screening of caregiver-accessible e-commerce platforms serving the UAE market. Physical sampling was conducted in Dubai across major retail settings (community pharmacies, supermarket/hypermarket chains, and oral-care retailers) during repeated visits within the sampling window. E-commerce sources were selected *a priori* based on their widespread use in the UAE, availability of national delivery, and the ability to view product listings and ingredient disclosures (e.g., large multi-vendor marketplaces and pharmacy/supermarket online stores). Products were included if they were explicitly marketed for children and displayed a complete ingredient list. Products lacking ingredient disclosure, not marketed for children, or representing duplicate formulations were excluded.

Products were categorized as conventionally marketed or natural/organic/herbal (NOHT) based on manufacturer labeling and consumer-facing descriptors. This classification reflects marketing claims rather than independently verified composition. All labeled ingredients were recorded verbatim and harmonized to preferred International Nomenclature of Cosmetic Ingredients (INCI) terms to address spelling variants and synonymous names.

### Evidence-mapping literature review

**Figure 1:**
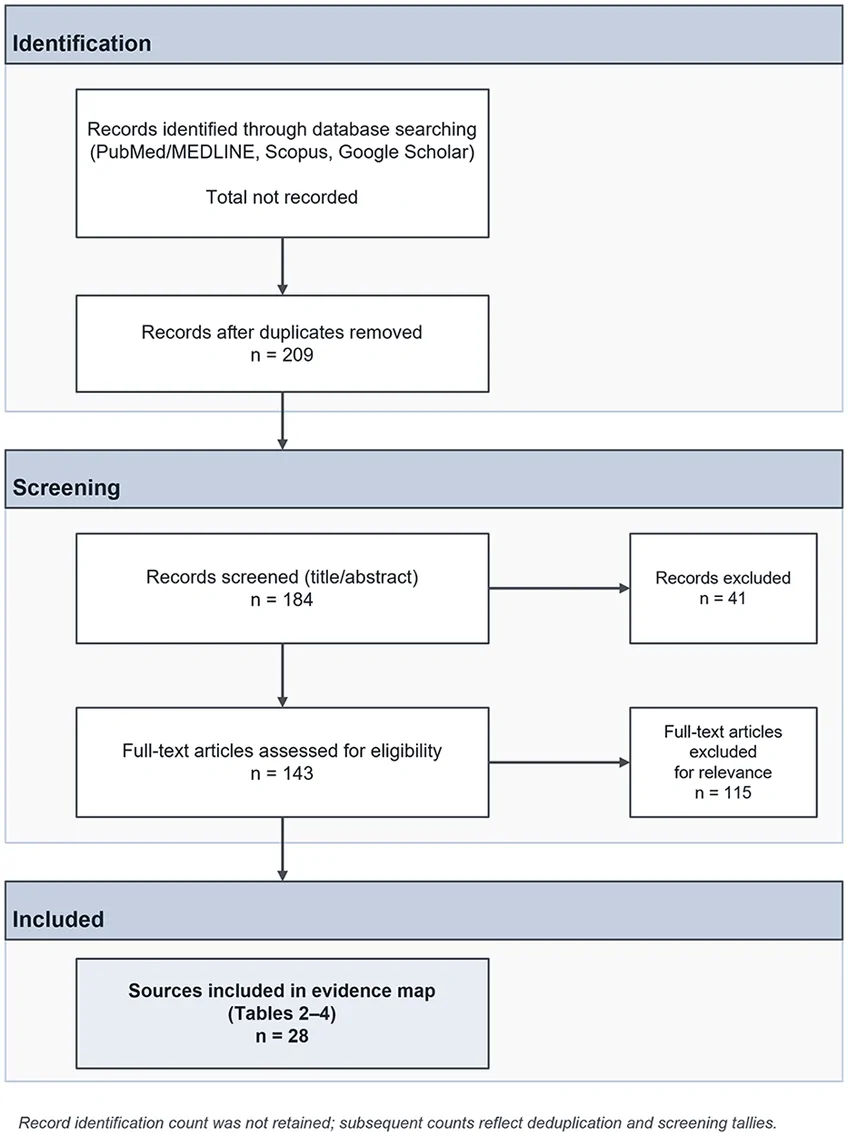
Evidence selection flow diagram (PRISMA-ScR style) for the evidence-mapping literature review. The number of records initially identified across databases was not retained; subsequent counts reflect deduplication and screening tallies (records after duplicates removed, *n* = 209; records screened, *n* = 184; full-text assessed, *n* = 143). The final included set corresponds to the unique sources cited in Tables 2, 3 (*n* = 28).

A structured evidence-mapping review was conducted using PubMed/MEDLINE, Scopus, and targeted Google Scholar searches. Searches were limited to English-language publications from January 2000 to 7 July 2026 (final search date). Database-specific search strategies combined ingredient names and synonyms with outcome- and exposure-related terms. Regulatory and authoritative sources were reviewed in parallel to identify formal safety assessments relevant to cosmetic and oral-care use (8, 13). To enhance coverage, reference lists of key reviews and authoritative assessments were also screened for eligible sources. Complete search logs (full database-specific strings, rerun dates, and database-level yields at identification) were not retained; however, deduplication and screening tallies from this workflow are summarized in Figure 1.

The evidence selection process and screening outcomes are summarized in Figure 1.

Search approach (evidence-informed narrative mapping): Searches were performed in PubMed/MEDLINE, Scopus, and Google Scholar and were limited to English-language records published from January 2000 to 7 July 2026 (final search date). For each ingredient (and common synonyms), database queries combined (i) the ingredient name block, (ii) outcome/safety terms, and (iii) toothpaste-relevant exposure/context terms, using Boolean operators and truncation where supported. A representative query structure was: (ingredient name OR synonym*) AND (toxicity OR “adverse effects” OR safety OR cytotoxic* OR irritation OR allergy* OR sensitization) AND (toothpaste OR dentifrice OR oral OR ingestion OR child* OR pediatric). Queries were translated to database-specific syntax [e.g., PubMed field tags such as (tiab), Scopus TITLE-ABS-KEY, and simplified keyword strings for Google Scholar] while retaining the same three-block logic; representative database-specific example strings are provided in the Supplementary Appendix.

### Eligibility criteria (evidence-mapping review)

#### Inclusion criteria

English-language publications (January 2000–7 July 2026) that reported any biological effect, toxicological endpoint, clinical outcome, or regulatory safety conclusion linked to at least one labeled toothpaste ingredient identified in the UAE market survey (Tables 2 and 3), including ingredient synonyms and preferred INCI terms. Eligible sources included regulatory/authoritative assessments, systematic reviews, human clinical or observational studies, case reports/series, animal studies, *in vitro* studies, and occupational exposure reports, provided that the ingredient and exposure context were explicitly described.

#### Exclusion criteria

Records were excluded if they did not pertain to an ingredient present on toothpaste labels in this survey; addressed unrelated chemical species or mixtures without ingredient-level attribution; lacked primary data or an actionable regulatory conclusion (e.g., narrative opinion pieces with no evaluable evidence) (9); or were non-English, outside the prespecified date range, or unavailable in full text. Studies were not excluded solely because the exposure context differed from routine toothpaste use; instead, non-dentifrice contexts were retained and coded in the evidence map using exposure route, dose relevance, and toothpaste relevance classifications.

### Screening, extraction, and verification

Records were screened in two stages (title/abstract and full-text) using predefined inclusion and exclusion criteria. Two reviewers independently screened records and extracted data using a standardized template. Disagreements at either stage were first discussed to clarify eligibility criteria and reconcile interpretation of exposure context; when uncertainty persisted, the senior author adjudicated to reach a final decision and ensure consistent application of the coding framework across ingredients. Inter-reviewer agreement statistics (e.g., percent agreement or Cohen's *κ*) were not calculated or retained. Regulatory assessments were identified and treated separately from primary experimental or clinical studies.

### Evidence hierarchy and classification

For each ingredient, reported findings were classified according to a predefined hierarchical framework: evidence source, exposure route, dose relevance relative to routine use, population relevance, evidence consistency, and relevance to routine pediatric toothpaste use. Hazard identification was defined as the presence of reported biological effects under specified conditions and was explicitly distinguished from clinical risk.

### Evidence tables

Evidence tables present ingredient-specific entries using preferred INCI names. Tables are divided into functional ingredients and sensory/plant-derived ingredients solely for readability, with identical classification criteria applied across both tables.

#### Ingredient prioritization for evidence mapping

The market survey yielded 190 unique labeled ingredients after deduplication (Table 1). Because mapping every ingredient was not feasible, Tables 2, 3 present an evidence map for a predefined prioritized subset selected using practical and relevance-based criteria: (i) ingredients frequently present across products in this UAE sample (high-frequency excipients or commonly repeated label entries); (ii) ingredients of heightened pediatric oral-exposure relevance (likely oral contact and partial ingestion during routine brushing); and/or (iii) ingredients with established regulatory interest or recurring safety discussion in authoritative assessments (e.g., preservatives, surfactants, colorants, fragrance/essential-oil components). This approach was intended to maximize interpretability for routine children's toothpaste use while maintaining a transparent scope. Each table entry was verified by at least two reviewers through independent cross-checking of ingredient identity, exposure route, and outcome coding against the source text.

**Table 1:** UAE market sample and labeled ingredient summary for children's toothpastes.

Metric	Conventional products	NOHT products	Overall/note
Products identified	42	23	65 total
Distinct labeled ingredients^a^	103	112	190 unique ingredients after deduplication
Shared ingredients	Not applicable	Not applicable	25 shared ingredients
Fluoride-containing products	35/42 (83.3%)	7/23 (30.4%)	42/65 overall (64.6%)
NOHT marketing descriptors	Not applicable	Natural, organic, herbal, fluoride-free, chemical-free	Reflects marketing claims only

a Distinct labeled ingredients refer to unique ingredient names recorded from product labels after harmonization of synonymous terms using preferred INCI nomenclature. Product counts and ingredient summaries are descriptive and relate only to products available through selected UAE retail sources during the sampling period; they do not represent the complete UAE market and do not imply product-level safety or risk.

**Table 2:** Evidence map of literature-reported biological effects associated with functional ingredients in children's toothpastes (hazard identification; not risk assessment).

Ingredient (INCI)	Functional class	Evidence source	Exposure route	Dose relevance^a^	Population relevance	Evidence consistency	Toothpaste relevance†	Key sources
Sodium fluoride	Active	Regulatory; human epidemiologic	Ingestion; oral	Routine-use range (context-dependent)	Children	Consistent	Direct	EFSA (14); pediatric fluoride guidance (3, 4)
Olaflur	Fluoride compound	Case report; regulatory	Oral contact; ingestion	Uncertain	Children/adults	Isolated	Direct	Cheilitis report (15); oral health monograph (16)
Hydrated silica	Abrasive	Human; experimental	Oral contact; ingestion	Routine-use range	Children/adults	Consistent	Direct	Dentifrice abrasivity review (17)
Calcium carbonate	Abrasive	Human; experimental	Oral contact; ingestion	Routine-use range	Children/adults	Consistent	Direct	Dentifrice abrasivity review (17)
Sodium lauryl sulfate	Surfactant	Human; *in-vitro*	Oral contact	Uncertain	Children/adults	Mixed	Direct	Cytotoxicity+scoping review (18, 19)
Cocamidopropyl betaine	Surfactant	Human; *in-vitro*	Oral contact; dermal	Uncertain	Children/adults	Mixed	Direct	*In-vitro* toothpaste ingredients (18)
Propylene glycol	Humectant	Human; case reports	Ingestion; systemic	Substantially above routine	Children	Isolated	Indirect	Pediatric toxicity review (20)
Carrageenan	Thickener	Animal; *in-vitro*	Ingestion	Substantially above routine	Animals/cells	Mixed	Indirect	Colonic cell inflammation (21)
Xanthan gum	Thickener	Occupational; experimental	Inhalation; ingestion	Substantially above routine	Adults	Isolated	Minimal	Xanthan production (22); worker symptoms (23)
Sodium benzoate	Preservative	Human; animal	Ingestion	Substantially above routine	Children/animals	Mixed	Indirect	Preservative challenge (24); rat study (25)
Phenoxyethanol	Preservative	Human; *in-vitro*	Oral contact; dermal	Uncertain	Children/adults	Mixed	Direct	Preservative apoptosis/necrosis (26)
Benzyl alcohol	Preservative	Case reports	Ingestion; systemic	Substantially above routine	Infants/children	Isolated	Indirect	Gasping syndrome report (27)
EDTA	Chelator	Experimental	Oral contact; systemic	Substantially above routine	Animals/cells	Isolated	Minimal	SCCS guidance (8)
Sorbitol	Sweetener	Human	Ingestion	Substantially above routine	Children/adults	Consistent	Indirect	Fructose-sorbitol malabsorption review (28)
Sucralose	Sweetener	Animal	Ingestion	Substantially above routine	Animals	Mixed	Indirect	Rodent neoplasia report (29)

a Dose relevance: relative to routine pediatric toothpaste use. †Toothpaste relevance: direct=dentifrice/oral-use evidence; indirect=non-dentifrice oral or consumer exposure; minimal=experimental/occupational context. Each row represents a single ingredient. Reported effects represent hazard identification only under specified study conditions and should not be interpreted as clinical harm or risk during routine pediatric toothpaste use. All entries were independently reviewed by at least two reviewers.

**Table 3:** Evidence map of literature-reported biological effects associated with sensory, colorant, and plant-derived ingredients in children's toothpastes (hazard identification; not risk assessment).

Ingredient (INCI)	Ingredient type	Evidence source	Exposure route	Dose relevance	Population relevance	Evidence consistency	Toothpaste relevance	Key sources
Titanium dioxide	Colorant	Animal; *in-vitro*	Ingestion	Substantially above routine	Animals/cells	Mixed	Indirect	Genotoxicity report (30)
Limonene	Fragrance component	Human	Oral contact; dermal	Uncertain	Children/adults	Consistent	Direct	Oxidized terpene allergy (31)
Linalool	Fragrance component	Human	Oral contact; dermal	Uncertain	Children/adults	Consistent	Direct	Oxidized terpene allergy (31)
Eugenol	Flavor agent	*In-vitro*; human	Oral contact	Uncertain	Children/adults	Mixed	Direct	Primary-tooth fibroblast toxicity (32)
Menthol	Flavor agent	Human; narrative reviews	Oral contact; inhalation	Uncertain	Children/adults	Mixed	Direct	Menthol narrative review (33)
Tea tree oil	Essential oil	Case report	Ingestion	Substantially above routine	Children	Isolated	Indirect	Pediatric ingestion case (34)
Eucalyptus oil	Essential oil	Case report	Ingestion	Substantially above routine	Children	Isolated	Indirect	Pediatric toxicity case (35)
Sage extract	Plant extract	Experimental	Ingestion	Substantially above routine	Animals	Isolated	Indirect	EMA/HMPC statement (36)
Myrrh extract	Plant extract	Animal	Systemic	Substantially above routine	Animals	Isolated	Minimal	Mouse toxicity study (37)
Fragaria vesca extract	Plant extract	Human	Oral contact	Uncertain	Susceptible individuals	Isolated	Direct	Fragaria vesca extract review (38)

Tables are divided by ingredient type for readability only. The same hierarchical classification criteria were applied across Tables 2, 3. Evidence reflects reported biological effects under specific exposure conditions and does not constitute product-level or ingredient-level risk assessment. All entries were independently reviewed by at least two reviewers.

### Data analysis

Descriptive analyses were used to summarize product characteristics and ingredient distribution. Statistical comparisons were limited to formulation features such as fluoride labeling and were interpreted descriptively rather than as indicators of safety or risk.

## Results

### Market characteristics and product classification

A total of 65 children's toothpastes were identified across selected UAE retail sources, comprising conventionally marketed products and those labeled as natural, organic, or herbal (NOHT). Summary characteristics of the market sample, including product counts, ingredient counts, category overlap, and fluoride labeling frequency, are presented in Table 1, which provides a descriptive overview of labeled composition without implying product-level safety or risk.

### Ingredient distribution and overlap between product categories

**Figure 2:**
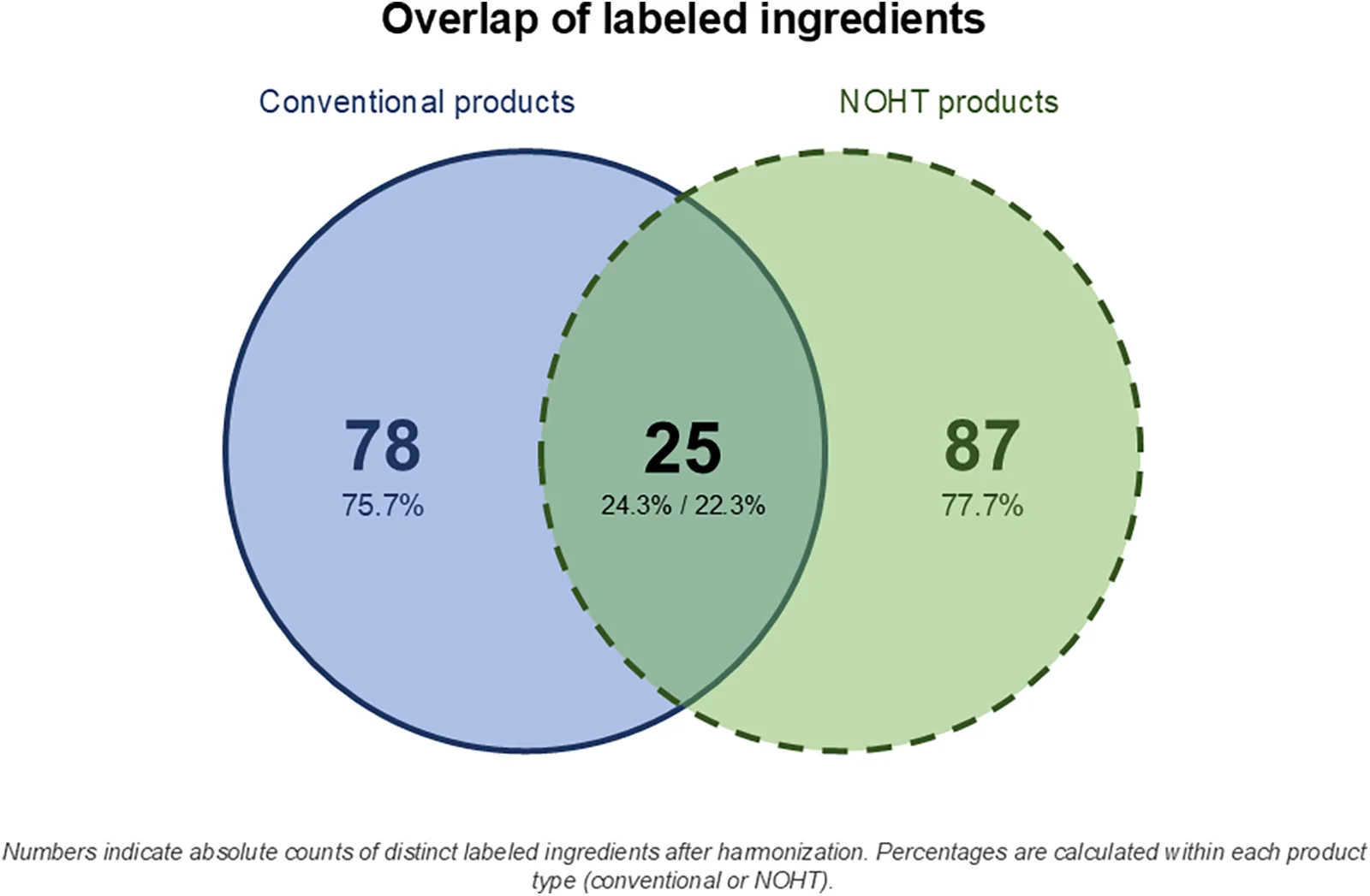
Overlap of labeled ingredients in conventional and natural/organic/herbal (NOHT) children's toothpastes identified in the UAE market. Venn diagram of distinct labeled ingredients in conventional (left) and natural/organic/herbal (NOHT; right) children's toothpastes after harmonizing synonymous names using preferred INCI terms. Numbers indicate conventional-only, NOHT-only, and shared ingredients. Overlap proportions use category-specific denominators: 24.3% = 25/103 (shared/distinct conventional) and 22.3% = 25/112 (shared/distinct NOHT).

Substantial overlap was observed between conventional and NOHT products in labeled ingredient classes, including surfactants, preservatives, humectants, and flavoring agents. As summarized in Table 1, both categories contained diverse ingredients and shared multiple excipients, indicating that marketing descriptors alone did not correspond to distinct ingredient profiles in this UAE sample. Ingredient overlap between categories is illustrated in Figure 2.

### Evidence mapping of literature-reported biological effects

The structured evidence map for functional ingredients is shown in Table 2. Each row represents a single ingredient and summarizes literature-reported biological effects classified by evidence source, exposure route, dose relevance relative to routine pediatric toothpaste use, population relevance, evidence consistency, and toothpaste relevance. Reported effects were derived from heterogeneous evidence sources, including regulatory assessments, human studies, animal models, and *in vitro* experiments, with many findings arising from exposure contexts that are not directly representative of routine pediatric dentifrice use.

The corresponding evidence map for sensory, colorant, and plant-derived ingredients is presented in Table 3. As in Table 2, evidence is coded using the same hierarchical framework to distinguish the origin and applicability of reported biological effects.

Across both evidence tables, reported effects varied widely in strength, consistency, and exposure context. Many findings arose from high-dose, experimental, occupational, or non-dentifrice scenarios and therefore have limited direct applicability to routine pediatric toothpaste use. Accordingly, Tables 2, 3 should be interpreted as a structured hazard-mapping summary rather than as estimates of ingredient-level or product-level risk.

Regulatory and labeling transparency considerations relevant to pediatric toothpaste marketing in the UAE and comparator frameworks are summarized in Table 4.

**Table 4:** Regulatory and labeling transparency considerations relevant to children's toothpaste marketed in the UAE.

Issue	UAE/GCC context	International comparator	Relevance to this study
Product classification	Children's toothpastes are generally regulated under cosmetics/personal care frameworks unless therapeutic claims are made; local requirements reference expectations for competent authority registration and labeling (12, 13, 39).	EU cosmetic regulation distinguishes cosmetic vs medicinal claims and sets general product obligations (40).	Influences the interpretation of safety wording and claim language in marketing and labeling.
Fluoride disclosure	A declaration is required when fluoride is present; labeling practices affect caregiver interpretation and professional advice (13, 39).	International pediatric guidance emphasizes age-appropriate use and supervision (3, 4).	Relevant to formulation differences observed between conventional and NOHT products in this sample.
Fragrance transparency	Generic “flavor/aroma” labeling remains common; GCC requirements address cosmetic labeling and safety expectations (39). Implementation timelines for UAE fragrance-allergen labeling have been reported (41).	The EU expanded allergen disclosure requirements for cosmetics (42).	Limits caregiver awareness of sensitizing components potentially present in children's toothpastes.
“Natural/organic” claims	Claims are regulated for truthfulness and must not mislead consumers (12); positioning terms are not standardized toxicological categories.	The EU common-criteria framework states claims must not imply superiority or safety without evidence (11).	Relevant to the interpretation of NOHT marketing and caregiver perceptions.
“Free-from” claims	Must not mislead consumers and should not denigrate permitted ingredients (12).	EU criteria discourage misleading “free-from” claims (11).	Relevant to caregiver perceptions when products are marketed as “chemical-free,” “fluoride-free,” or similar.
Pediatric safety wording	Label accuracy and required warnings are part of cosmetic labeling expectations; supervision messaging is particularly important for young children who may ingest toothpaste (13, 39).	International guidance emphasizes adult supervision and age-appropriate amounts (3, 4).	Critical for interpreting ingestion risk and aligning labeling with caregiver use conditions.

## Discussion

Two findings are central. First, after accounting for ingredient overlap between categories, the deduplicated dataset included 190 unique labeled ingredients (Table 1). Second, within the evidence map, the mapped biological effects varied widely in exposure context and dose relevance (Tables 2 and 3), underscoring the need for exposure-aware interpretation rather than label-based safety inference.

In this context, children represent a unique exposure group because of partial ingestion, developing metabolic pathways, and heightened susceptibility during key developmental windows, reinforcing the need to interpret the evidence map in relation to the likely dose, route, and frequency of exposure during routine brushing (5, 9).

Against this exposure-aware backdrop, Table 4 provides context for interpreting these findings in relation to ingredient disclosure and marketing language. Relevant regulatory and policy frameworks emphasize that labeling conventions and marketing claims should be interpreted in light of transparency requirements and evidence standards for claim substantiation. A recurring limitation is that ingredient lists rarely provide quantitative information (e.g., concentrations), which limits interpretation of exposures and undermines the comparability implied by marketing claims. In particular, the continued use of generic terms such as “flavor/aroma,” the evolving requirements for fragrance-allergen transparency, and the regulatory expectations that “natural,” “organic,” or “free-from” claims should not be misleading underscore why NOHT descriptors cannot be treated as proxies for reduced hazard or safety in the absence of concentration and exposure information (11, 12, 19, 39–42).

Some safety considerations relate to impurity control and manufacturing quality (e.g., contaminants or batch-to-batch variability) rather than an ingredient's intrinsic hazard, further emphasizing that label-based ingredient lists cannot fully represent exposure-relevant product safety without concentration, purity, and quality data (8, 9).

Consistent with these labeling limitations, the observed market-level patterns align with published formulation reviews and evidence-mapping frameworks that emphasize exposure context when interpreting reported biological effects. Notably, NOHT positioning did not uniformly correspond to fluoride exclusion in this sample (Table 1), reinforcing that marketing categories do not map reliably onto functional formulation features. Given the mix of multinational brands, regional distributors, and e-commerce supply chains represented in UAE retail availability, labeling detail and claim language may vary across batches and distributors, highlighting the value of periodic surveillance of pediatric oral-care products. These UAE market observations are consistent with prior analyses noting that children's toothpaste formulations commonly share multiple excipients across brands and that “natural” vs. “conventional” positioning does not necessarily translate into distinct ingredient profiles, while the interpretability of reported biological effects depends strongly on exposure context (7, 8).

Overall, the evidence map highlights substantial variability in source type, exposure route, and dose relevance (Tables 2 and 3). Because many findings derive from high-dose or non-dentifrice contexts, they should not be directly extrapolated to routine pediatric toothpaste use. Consistent with cosmetic safety frameworks, we present these findings as hazard identification within an evidence-mapping approach rather than exposure-based risk characterization (8, 9).

Future work should pair market surveys with laboratory verification of ingredient identity and concentration and integrate age-specific exposure parameters (e.g., typical toothpaste mass used and swallowing fractions) to enable risk-context interpretation beyond hazard mapping.

Fluoride labeling patterns observed in this sample reflect formulation differences relevant to caregiver choice and professional guidance but should not be interpreted as a comparative safety assessment. Established clinical recommendations and regulatory risk assessments consistently frame fluoride benefit and safety in terms of dose, frequency, and supervised use in young children. Current pediatric guidance and risk assessments emphasize age-appropriate amounts, supervision, and the context of exposure (3–5, 14).

More broadly, these considerations support prioritizing evidence-based fluoride guidance, age-appropriate quantities, and adult supervision, while encouraging clearer disclosure of fragrance components and more rigorous substantiation of “natural,” “organic,” and “free-from” claims to reduce misinterpretation of labeling as a proxy for safety (3–5, 11, 12, 14, 19, 39–42).

### Limitations

This study relied on product labeling without laboratory verification of ingredient identity, concentration, or labeling accuracy, and some labels used generic descriptors (e.g., “flavor/aroma”) that may limit ingredient-level transparency. NOHT categorization was based on consumer-facing marketing claims and was not independently verified against certification standards. The evidence map does not substitute for quantitative exposure assessment, dose–response evaluation, formal risk-of-bias grading, or clinical safety evaluation; accordingly, reported biological effects should be interpreted as hazard identification rather than as evidence of clinical risk. **Search transparency:** complete database-level search logs (full database-specific strings, rerun dates, and identification-stage yields) were not retained, which limits reproducibility; however, deduplication and screening tallies from this workflow are reported in Figure 1. **Screening reliability:** formal inter-reviewer agreement statistics (e.g., percent agreement or Cohen's *κ*) were not calculated, limiting quantitative assessment of screening consistency. Products reflect selected UAE retail sources during the sampling window and may not represent the complete UAE market or changes in product availability over time.

## Conclusions

In this UAE market sample, children's toothpastes marketed as conventional or natural/organic/herbal shared diverse ingredients, and fluoride labeling was more common among conventional products in this sample (a descriptive formulation and labeling pattern, not a safety comparison). These findings represent structured hazard identification and evidence mapping rather than product-level safety assessment. Ingredient concentration and exposure data are essential for evaluating clinical risk, and marketing descriptors such as “natural” or “herbal” should not be used as proxies for safety.

## Data Availability

The original contributions presented in the study are included in the article/Supplementary Material, further inquiries can be directed to the corresponding author.

## Appendix: Database-specific search strategy examples

The following database-specific strings are representative templates illustrating the three-block query logic applied across databases: (1) ingredient/synonyms, (2) outcome/safety terms, and (3) toothpaste/exposure context terms. Searches were limited to English-language records published from January 2000 through 7 July 2026 (final search date). Exact rerun logs and record counts were not retained; therefore, the strings below are provided as reproducible examples of the query structure and database syntax used. Search terms reflect indexed terminology (including occasional use of keywords such as “risk”) and do not imply that this study performed a risk assessment.

**Core term blocks.** Outcome/safety block: toxicity OR “adverse effects” OR safety OR cytotoxic* OR irritation OR allerg* OR sensitization. Toothpaste/exposure context block: toothpaste OR dentifrice OR oral OR ingestion OR child* OR pediatric. Ingredient blocks were customized per ingredient using preferred INCI names and common abbreviations/synonyms (e.g., “sodium lauryl sulfate” OR SLS).

**Filters/limits applied:** English language; publication date January 2000–7 July 2026 (final search date); queries executed using title/abstract field tags ([tiab]) where applicable.

**PubMed/MEDLINE (example strings using field tags):**

1) Sodium lauryl sulfate (SLS): ((“sodium lauryl sulfate"[tiab] OR SLS[tiab]) AND (toxicity[tiab] OR “adverse effects"[tiab] OR safety[tiab] OR cytotoxic*[tiab] OR irritation[tiab] OR allerg*[tiab] OR sensitization[tiab]) AND (toothpaste[tiab] OR dentifrice[tiab] OR oral[tiab] OR ingestion[tiab] OR child*[tiab] OR pediatric[tiab]))

2) Titanium dioxide (TiO2): ((“titanium dioxide"[tiab] OR TiO2[tiab]) AND (toxicity[tiab] OR “adverse effects"[tiab] OR safety[tiab] OR cytotoxic*[tiab] OR irritation[tiab] OR allerg*[tiab] OR sensitization[tiab] OR genotoxic*[tiab]) AND (toothpaste[tiab] OR dentifrice[tiab] OR oral[tiab] OR ingestion[tiab] OR child*[tiab] OR pediatric[tiab]))

3) Fluoride (sodium fluoride OR stannous fluoride): ((“sodium fluoride"[tiab] OR “stannous fluoride"[tiab] OR fluoride[tiab]) AND (risk[tiab] OR safety[tiab] OR toxicity[tiab] OR fluorosis[tiab] OR ingestion[tiab]) AND (toothpaste[tiab] OR dentifrice[tiab] OR child*[tiab] OR pediatric[tiab]))

4) Limonene: ((limonene[tiab]) AND (toxicity[tiab] OR “adverse effects"[tiab] OR safety[tiab] OR irritation[tiab] OR allerg*[tiab] OR sensitization[tiab]) AND (toothpaste[tiab] OR dentifrice[tiab] OR oral[tiab] OR child*[tiab] OR pediatric[tiab]))

5) Linalool: ((linalool[tiab]) AND (toxicity[tiab] OR “adverse effects"[tiab] OR safety[tiab] OR irritation[tiab] OR allerg*[tiab] OR sensitization[tiab]) AND (toothpaste[tiab] OR dentifrice[tiab] OR oral[tiab] OR child*[tiab] OR pediatric[tiab]))

6) Propylene glycol: ((“propylene glycol"[tiab]) AND (toxicity[tiab] OR “adverse effects"[tiab] OR safety[tiab] OR irritation[tiab] OR allerg*[tiab] OR sensitization[tiab] OR ingestion[tiab]) AND (toothpaste[tiab] OR dentifrice[tiab] OR oral[tiab] OR ingestion[tiab] OR child*[tiab] OR pediatric[tiab]))

7) Carrageenan: ((carrageenan[tiab]) AND (toxicity[tiab] OR “adverse effects"[tiab] OR safety[tiab] OR cytotoxic*[tiab] OR inflammation[tiab]) AND (toothpaste[tiab] OR dentifrice[tiab] OR oral[tiab] OR ingestion[tiab] OR child*[tiab] OR pediatric[tiab])).

**Filters/limits applied:** English language; publication year range 2000–2026 (through 7 July 2026); searches run in TITLE-ABS-KEY fields.

**Scopus (example strings using TITLE-ABS-KEY):**

1) Sodium lauryl sulfate (SLS): TITLE-ABS-KEY ((“sodium lauryl sulfate” OR SLS) AND (toxicity OR “adverse effects” OR safety OR cytotoxic* OR irritation OR allerg* OR sensitization) AND (toothpaste OR dentifrice OR oral OR ingestion OR child* OR pediatric))

2) Titanium dioxide (TiO2): TITLE-ABS-KEY ((“titanium dioxide” OR TiO2) AND (toxicity OR “adverse effects” OR safety OR cytotoxic* OR irritation OR allerg* OR sensitization OR genotoxic*) AND (toothpaste OR dentifrice OR oral OR ingestion OR child* OR pediatric))

3) Fluoride: TITLE-ABS-KEY ((“sodium fluoride” OR “stannous fluoride” OR fluoride) AND (risk OR safety OR toxicity OR fluorosis OR ingestion) AND (toothpaste OR dentifrice OR child* OR pediatric))

4) Limonene: TITLE-ABS-KEY ((limonene) AND (toxicity OR “adverse effects” OR safety OR irritation OR allerg* OR sensitization) AND (toothpaste OR dentifrice OR oral OR child* OR pediatric))

5) Linalool: TITLE-ABS-KEY ((linalool) AND (toxicity OR “adverse effects” OR safety OR irritation OR allerg* OR sensitization) AND (toothpaste OR dentifrice OR oral OR child* OR pediatric))

6) Propylene glycol: TITLE-ABS-KEY ((“propylene glycol”) AND (toxicity OR “adverse effects” OR safety OR irritation OR allerg* OR sensitization OR ingestion) AND (toothpaste OR dentifrice OR oral OR ingestion OR child* OR pediatric))

7) Carrageenan: TITLE-ABS-KEY ((carrageenan) AND (toxicity OR “adverse effects” OR safety OR cytotoxic* OR inflammation) AND (toothpaste OR dentifrice OR oral OR ingestion OR child* OR pediatric)).

**Filters/limits applied:** Custom date range January 2000–7 July 2,026 and relevance sorting (where supported); English-language screening applied during eligibility review.

**Screening approach:** Results were reviewed iteratively in relevance order; screening continued until successive pages yielded no new eligible sources for the ingredient/exposure context of interest.

**Google Scholar (example strings; simplified keyword syntax):**

1) “sodium lauryl sulfate” OR SLS toxicity “adverse effects” safety cytotoxic toothpaste dentifrice oral ingestion child pediatric

2) “titanium dioxide” OR TiO2 genotoxicity toxicity toothpaste dentifrice ingestion child pediatric

3) “sodium fluoride” OR “stannous fluoride” fluorosis safety risk toothpaste dentifrice child pediatric

4) limonene allergy sensitization irritation toothpaste dentifrice child pediatric

5) linalool allergy sensitization irritation toothpaste dentifrice child pediatric

6) “propylene glycol” toxicity ingestion child pediatric toothpaste dentifrice

7) carrageenan inflammation cytotoxicity ingestion toothpaste dentifrice child pediatric

Results were screened for relevance to the ingredient of interest and to biological/toxicological effects, with emphasis on identifying additional eligible sources not retrieved in indexed databases.
