# Supplementary material for: Labeled ingredient composition and literature-reported hazard signals in children's toothpastes available through selected UAE retail sources
Source: Front Oral Health. 2026 Aug 26;7:1932715. doi: 10.3389/froh.2026.1932715 (PMC13553250; doi:10.3389/froh.2026.1932715)
Supplement: Supplementary file 1 [file Table1.docx]

**APPENDIX 1 Children’s Regular Toothpastes in the UAE Market with a Description of their Ingredients**

| **Toothpaste** | **Ingredients** | **Type of ingredient** | **Purpose** |
| --- | --- | --- | --- |
| **Signal Kids Strawberry** | Sodium Fluoride | Active ingredient | Anti-caries activity, whitening effects, halitosis control |
|  | Aqua (Water) | Solvents | Solvent |
|  | Sorbitol | Inactive - Sweeteners | Substitute for sugar used as a sweetening agent |
|  | Hydrated Silica | Whitening/plaque removal agents | Used as an abrasive, active in extrinsic stain and plaque removal |
|  | PEG-32 | Surfactant | Act as penetration enhancers |
|  | SLS | Emulsifier | Thickening and foaming agent. Antimicrobial properties |
|  | Cellulose gum | Thickening agents | Thickening agent |
|  | Aroma | Inactive - Fragrances | Fragrance additive |
|  | Sodium Saccharine | Inactive - Sweeteners | Artificial sweetener |
|  | Limonene | Inactive - Fragrances | Flavor and fragrance additive |
|  | Phenylcarbinol | Solvents | Solvent |
|  | CI 73360: D&C Red 30 | Inactive - Artificial colorant | Colorant |
| **Oral-B BABY 0-2 YEARS** | Sodium Fluoride 500 ppm | Active ingredient | Anti-caries activity, whitening effects, halitosis control |
|  | Sorbitol | Inactive - Sweeteners | Substitute for sugar used as a sweetening agent |
|  | Aqua | Solvents | Solvent |
|  | Hydrated Silica | Whitening/plaque removal agents | Used as an abrasive, active in extrinsic stain and plaque removal |
|  | Sodium Lauryl Sulfate | Emulsifier | Thickening and foaming agent. Antimicrobial properties |
|  | Trisodium Phosphate | Whitening/plaque removal agents | Not found |
|  | Cellulose Gum | Thickening agents | Thickening agent |
|  | Aroma | Inactive - Fragrances | Fragrance additive |
|  | Sodium Phosphate | Emulsifier | Emulsifier |
|  | Sodium Saccharin | Inactive - Sweeteners | Artificial sweetener |
|  | Limonene | Inactive - Fragrances | Flavor and fragrance additive |
|  | Benzyl Alcohol | Solvents | Solvent |
|  | Sodium Hydroxide | Buffering agent | Buffering agent |
|  | CI 42090 - Brilliant Blue FCF (Blue 1) | Inactive - Artificial colorant | Colorant |
| **Colgate 2-5 Years Bubble Fruit Flavor** | **Sorbitol** | Inactive - Sweeteners | Substitute for sugar used as a sweetening agent |
|  | Aqua | Solvents | Solvent |
|  | **Hydrated Silica** | Whitening/plaque removal agents | Used as an abrasive, active in extrinsic stain and plaque removal |
|  | **PEG-12** | Surfactant | Act as penetration enhancers |
|  | **Sodium Lauryl Sulfate** | Emulsifier | Thickening and foaming agent. Antimicrobial properties |
|  | **Aroma** | Inactive - Fragrances | Fragrance additive |
|  | **Cellulose gum** | Thickening agents | Thickening agent |
|  | **Tetrasodium Pyrophosphate** | Chelating agent | Anti-calculus and whitening agent |
|  | **Sodium Saccharin** | Inactive - Sweeteners | Artificial sweetener |
|  | **Sodium Fluoride 500 ppm** | Active ingredient | Anti-caries activity, whitening effects, halitosis control |
|  | **Cinnamal** | Inactive - Fragrances | Fragrance additive |
|  | **Eugenol** | Inactive - Fragrances | Flavor and fragrance additive |
|  | **Limonene** | Inactive - Fragrances | Flavor and fragrance additive |
|  | **Cl 42090** | Inactive - Artificial colorant | Colorant |
| **Colgate 6+ minions** | Sodium Fluoride 1450 ppm | Active ingredient | Anti-caries activity, whitening effects, halitosis control |
|  | Sorbitol | Inactive - Sweeteners | Substitute for sugar used as a sweetening agent |
|  | Aqua | Solvents | Solvent |
|  | Hydrated Silica | Whitening/plaque removal agents | Used as an abrasive, active in extrinsic stain and plaque removal |
|  | PEG-12 | Surfactant | Act as penetration enhancers |
|  | Xanthan Gum | Thickening agents | Thickening and stabilizing agent |
|  | Sodium Lauryl Sulphate | Emulsifier | Thickening and foaming agent. Antimicrobial properties |
|  | Sodium Saccharin | Inactive - Sweeteners | Artificial sweetener |
|  | Aroma | Inactive - Fragrances | Fragrance additive |
|  | Hydroxypropyl Methylcellulose | Surfactant | Foaming and enhancing creaminess |
|  | CI 73360 D&C Red 30 | Inactive - Artificial colorant | Colorant |
|  | CI 74160 | Inactive - Artificial colorant | Colorant |
| **Colgate 6+ barbie** | Sodium Fluoride 1450 ppm | Active ingredient | Anti-caries activity, whitening effects, halitosis control |
|  | Sorbitol | Inactive - Sweeteners | Substitute for sugar used as a sweetening agent |
|  | Aqua | Solvents | Solvent |
|  | Hydrated Silica | Whitening/plaque removal agents | Used as an abrasive, active in extrinsic stain and plaque removal |
|  | Xylitol | Whitening/plaque removal agents | Active in caries prevention + Effective against *S. mutans* |
|  | PEG-12 | Surfactant | Act as penetration enhancers |
|  | Cellulose Gum | Thickening agents | Thickening agent |
|  | Benzyl Alcohol | Solvents | Solvent |
|  | Sodium lauryl Sulphate | Emulsifier | Thickening and foaming agent. Antimicrobial properties |
|  | Aroma | Inactive - Fragrances | Fragrance additive |
| **Colgate 6+ batman** | Sodium Fluoride 1450 ppm | Active ingredient | Anti-caries activity, whitening effects, halitosis control |
|  | Sorbitol | Inactive - Sweeteners | Substitute for sugar used as a sweetening agent |
|  | Aqua | Solvents | Solvent |
|  | Hydrated Silica | Whitening/plaque removal agents | Used as an abrasive, active in extrinsic stain and plaque removal |
|  | Xylitol | Whitening/plaque removal agents | Active in caries prevention + Effective against S. mutans |
|  | PEG-12 | Surfactant | Act as penetration enhancers |
|  | Cellulose Gum | Thickening agents | Thickening agent |
|  | Benzyl Alcohol | Solvents | Solvent |
|  | Sodium lauryl Sulphate | Emulsifier | Thickening and foaming agent. Antimicrobial properties |
|  | Aroma | Inactive - Fragrances | Fragrance additive |
| **Jordan Kids 0-5 years Vegan** | Sodium Fluoride 500 ppm | Active ingredient | Anti-caries activity, whitening effects, halitosis control |
|  | Sorbitol | Inactive - Sweeteners | Substitute for sugar used as a sweetening agent |
|  | Aqua | Solvents | Solvent |
|  | Hydrated Silica | Whitening/plaque removal agents | Used as an abrasive, active in extrinsic stain and plaque removal |
|  | Glycerin | Wetting agents | Used to prevent toothpaste from drying out |
|  | PEG-32 | Surfactant | Act as penetration enhancers |
|  | Cocamidopropyl Betaine | Surfactant | Thickening and foaming agent |
|  | Trisodium Phosphate | Whitening/plaque removal agents | Not found |
|  | Cellulose Gum | Thickening agents | Thickening agent |
|  | Sodium Saccharin | Inactive - Sweeteners | Artificial sweetener |
|  | Sodium Benzoate | Preserving agents | Preserving agent |
|  | Sodium Sulphate | Preserving agents | Preserving agent |
|  | Sodium Chloride | Abrasives | Abrasive agent |
|  | Aroma | Inactive - Fragrances | Fragrance additive |
|  | CI 42051 | Inactive - Artificial colorant | Colorant |
| **Jordan Junior 6-12 years** | Sodium Fluoride 1450 ppm | Active ingredient | Anti-caries activity, whitening effects, halitosis control |
|  | Sorbitol | Inactive - Sweeteners | Substitute for sugar used as a sweetening agent |
|  | Aqua | Solvents | Solvent |
|  | Hydrated Silica | Whitening/plaque removal agents | Used as an abrasive, active in extrinsic stain and plaque removal |
|  | Glycerin | Wetting agents | Used to prevent toothpaste from drying out |
|  | PEG-32 | Surfactant | Act as penetration enhancers |
|  | Trisodium Phosphate | Whitening/plaque removal agents | Not found |
|  | Cellulose Gum | Thickening agents | Thickening agent |
|  | Cocamidopropyl Betaine | Surfactant | Thickening and foaming agent |
|  | Sodium Benzoate | Preserving agents | Preserving agent |
|  | Sodium Chloride | Abrasives | Abrasive agent |
|  | Sodium Sulfate | Preserving agents | Preserving agent |
|  | Sodium Saccharin | Inactive - Sweeteners | Artificial sweetener |
|  | CI 42051 | Inactive - Artificial colorant | Colorant |
|  | CI 74160 | Inactive - Artificial colorant | Colorant |
|  | Aroma | Inactive - Fragrances | Fragrance additive |
| **Crest KID'S FLUORIDE ANTICAVITY TOOTHPASTE STRAWBERRY FLAVOR 2+** | Sodium Fluoride 0.24% | Active ingredient | Anti-caries activity, whitening effects, halitosis control |
|  | Sorbitol | Inactive - Sweeteners | Substitute for sugar used as a sweetening agent |
|  | Water | Solvents | Solvent |
|  | Hydrated Silica | Whitening/plaque removal agents | Used as an abrasive, active in extrinsic stain and plaque removal |
|  | Sodium Lauryl Sulfate | Emulsifier | Thickening and foaming agent. Antimicrobial properties |
|  | Trisodium Phosphate | Whitening/plaque removal agents | Not found |
|  | Sodium Phosphate | Emulsifier | Emulsifier |
|  | Cellulose Gum | Thickening agents | Thickening agent |
|  | Flavor | Inactive - Flavorings | Flavoring agent |
|  | Sodium Saccharin | Inactive - Sweeteners | Artificial sweetener |
|  | Carbomer | Inactive - Emulsifier | Binder (thickener) |
|  | Red 40 | Inactive - Artificial colorant | Colorant |
| **Hello Kitty Toothpaste Strawberry** | Sodium Fluoride 1000 ppm | Active ingredient | Anti-caries activity, whitening effects, halitosis control |
|  | Sorbitol | Inactive - Sweeteners | Substitute for sugar used as a sweetening agent |
|  | Water | Solvents | Solvent |
|  | Hydrated Silica | Whitening/plaque removal agents | Used as an abrasive, active in extrinsic stain and plaque removal |
|  | Sodium Lauryl Sulfate | Emulsifier | Thickening and foaming agent. Antimicrobial properties |
|  | PEG-32 | Surfactant | Act as penetration enhancers |
|  | Cellulose Gum | Thickening agents | Thickening agent |
|  | Parfum | Inactive - Fragrances | Fragrance additive |
|  | CI 77891 | Artificial - Colorant + Whitening/plaque removal agents | Active in extrinsic stain removal |
|  | Sodium Saccharin | Inactive - Sweeteners | Artificial sweetener |
|  | Trisodium Phosphate | Whitening/plaque removal agents | Not found |
|  | CI 73360 ● D&C Red 30 | Inactive - Artificial colorant | Colorant |
| **Biorepair Oral Care Kids 0-6 Age Strawberry Flavor** | Aqua | Solvents | Solvent |
|  | Glycerin | Wetting agents | Used to prevent toothpaste from drying out |
|  | Zinc Hydroxyapatite | Active ingredient | Microrepair |
|  | Sorbitol | Inactive - Sweeteners | Substitute for sugar used as a sweetening agent |
|  | PEG-32 | Surfactant | Act as penetration enhancers |
|  | Silica | Abrasives | Abrasive agent |
|  | Cellulose gum | Thickening agents | Thickening agent |
|  | Aroma | Inactive - Fragrances | Fragrance additive |
|  | Sodium Myristoyl Sarcosinate | Emulsifier | pH buffering agent |
|  | Sodium Methyl Cocoyl Taurate | Surfactant | Foaimng agent |
|  | Fragaria Vesca Juice | Vegetable-based ingredients | Contains various biologically active phenolic compounds like tannins, anthocyanins, flavonoids, and phenolic acids. These compounds contribute to its antioxidant, antimicrobial, anti-inflammatory, anticoagulant, and vasodilatory effects |
|  | Mentha Piperita Oil | Inactive - Flavorings | Functions as a flavoring and scent for a toothpaste |
|  | Sodium Saccharin | Inactive - Sweeteners | Artificial sweetener |
|  | Citric Acid | Whitening/plaque removal agents | Active in dental plaque removal and whitening teeth |
|  | Sodium Benzoate | Preserving agents | Preserving agent |
|  | Potassium Sorbate | Preserving agents | Preserving agent |
|  | Phenoxyethanol | Preserving agents | Preserving agent |
|  | Benzyl Alcohol | Solvents | Solvent |
|  | Anethole | Inactive - Flavorings and fragrance | Flavor and fragrance additive |
|  | Menthol | Inactive - Flavorings | Functions as a flavoring and scent for a toothpaste |
| **Biorepair Oral Care Kids 0-6 Age Peach Flavor** | Aqua | Solvents | Solvent |
|  | Glycerin | Wetting agents | Used to prevent toothpaste from drying out |
|  | Zinc Hydroxyapatite | Active ingredient | Microrepair |
|  | Sorbitol | Inactive - Sweeteners | Substitute for sugar used as a sweetening agent |
|  | PEG-32 | Surfactant | Act as penetration enhancers |
|  | Silica | Abrasives | Abrasive agent |
|  | Cellulose gum | Thickening agents | Thickening agent |
|  | Aroma | Inactive - Fragrances | Fragrance additive |
|  | Sodium Myristoyl Sarcosinate | Emulsifier | pH buffering agent |
|  | Sodium Methyl Cocoyl Taurate | Surfactant | Foaimng agent |
|  | Prunus Persica Juice | Inactive - Flavorings | Flavor and fragrance additive |
|  | Sodium Saccharin | Inactive - Sweeteners | Artificial sweetener |
|  | Citric Acid | Whitening/plaque removal agents | Active in dental plaque removal and whitening teeth |
|  | Sodium Benzoate | Preserving agents | Preserving agent |
|  | Potassium Sorbate | Preserving agents | Preserving agent |
|  | Phenoxyethanol | Preserving agents | Preserving agent |
|  | Benzyl Alcohol | Solvents | Solvent |
| **Theramed 2 in 1 Junior Strawberry Taste Toothpaste + Mouthwash** | Sodium Fluoride 1450 ppm | Active ingredient | Anti-caries activity, whitening effects, halitosis control |
|  | Aqua | Solvents | Solvent |
|  | Sorbitol | Inactive - Sweeteners | Substitute for sugar used as a sweetening agent |
|  | Hydrated Silica | Whitening/plaque removal agents | Used as an abrasive, active in extrinsic stain and plaque removal |
|  | PEG-8 | Surfactant | Act as penetration enhancers |
|  | Xanthan Gum | Thickening agents | Thickening and stabilizing agent |
|  | Cocamidopropyl Betaine | Surfactant | Thickening and foaming agent |
|  | PEG-30 | Surfactant | Act as penetration enhancers |
|  | Glyceryl Stearate | Emulsifier | Moisturizer and binder |
|  | Sodium Saccharin | Inactive - Sweeteners | Artificial sweetener |
|  | Disodium Phosphate | Preservative and a stabilizer | Stabilises dental products and controls acidity |
|  | Calcium Glycerophosphate | Remineralization agents | Enhances the effects of F on enamel de- and re-mineralization processes. Reduced enamel erosion |
|  | Sodium Chloride | Abrasives | Abrasive agent |
|  | Methylparaben | Preserving agents | Preserving agent |
|  | Sodium Benzoate | Preserving agents | Preserving agent |
|  | Aroma | Inactive - Fragrances | Fragrance additive |
|  | CI 14720 | Inactive - Artificial colorant | Colorant |
| **Aquafresh Milk Teeth 0-2 Years** | Sodium Fluoride 500 ppm | Active ingredient | Anti-caries activity, whitening effects, halitosis control |
|  | Aqua | Solvents | Solvent |
|  | Hydrated Silica | Whitening/plaque removal agents | Used as an abrasive, active in extrinsic stain and plaque removal |
|  | Sorbitol | Inactive - Sweeteners | Substitute for sugar used as a sweetening agent |
|  | Glycerin | Wetting agents | Used to prevent toothpaste from drying out |
|  | Xanthan Gum | Thickening agents | Thickening and stabilizing agent |
|  | Titanium Dioxide | Artificial - Colorant + Whitening/plaque removal agents | Active in extrinsic stain removal |
|  | Aroma | Inactive - Fragrances | Fragrance additive |
|  | Sodium Saccharin | Inactive - Sweeteners | Artificial sweetener |
|  | Cocamidopropyl Betaine | Surfactant | Thickening and foaming agent |
|  | Sodium Methyl Cocoyl Taurate | Surfactant | Foaimng agent |
| **Aquafresh Little Teeth 3-5 Years** | Sodium Fluoride 500 ppm | Active ingredient | Anti-caries activity, whitening effects, halitosis control |
|  | Aqua | Solvents | Solvent |
|  | Hydrated Silica | Whitening/plaque removal agents | Used as an abrasive, active in extrinsic stain and plaque removal |
|  | Sorbitol | Inactive - Sweeteners | Substitute for sugar used as a sweetening agent |
|  | Glycerin | Wetting agents | Used to prevent toothpaste from drying out |
|  | Xanthan Gum | Thickening agents | Thickening and stabilizing agent |
|  | Titanium Dioxide | Artificial - Colorant + Whitening/plaque removal agents | Active in extrinsic stain removal |
|  | Aroma | Inactive - Fragrances | Fragrance additive |
|  | Sodium Saccharin | Inactive - Sweeteners | Artificial sweetener |
|  | Cocamidopropyl Betaine | Surfactant | Thickening and foaming agent |
|  | Sodium Methyl Cocoyl Taurate | Surfactant | Foaimng agent |
|  | Limonene | Inactive - Fragrances | Flavor and fragrance additive |
|  | CI 73360 D&C Red 30 | Inactive - Artificial colorant | Colorant |
|  | CI 74160 | Inactive - Artificial colorant | Colorant |
| **Aquafresh My Big Teeth 6+ Years** | Sodium Fluoride 1450 ppm | Active ingredient | Anti-caries activity, whitening effects, halitosis control |
|  | Aqua | Solvents | Solvent |
|  | Hydrated Silica | Whitening/plaque removal agents | Used as an abrasive, active in extrinsic stain and plaque removal |
|  | Sorbitol | Inactive - Sweeteners | Substitute for sugar used as a sweetening agent |
|  | Glycerin | Wetting agents | Used to prevent toothpaste from drying out |
|  | Xanthan Gum | Thickening agents | Thickening and stabilizing agent |
|  | Titanium Dioxide | Artificial - Colorant + Whitening/plaque removal agents | Active in extrinsic stain removal |
|  | Aroma | Inactive - Fragrances | Fragrance additive |
|  | Sodium Saccharin | Inactive - Sweeteners | Artificial sweetener |
|  | Cocamidopropyl Betaine | Surfactant | Thickening and foaming agent |
|  | Sodium Methyl Cocoyl Taurate | Surfactant | Foaimng agent |
|  | Limonene | Inactive - Fragrances | Flavor and fragrance additive |
|  | CI 73360 D&C Red 30 | Inactive - Artificial colorant | Colorant |
|  | CI 74160 | Inactive - Artificial colorant | Colorant |
|  | Chondrus Crispus (Carrageenan) | Thickening agents | Thickening agent |
| **Boots Kids Gentle Mint Toothpaste Ages 0-2** | Sodium Fluoride 1000 ppm | Active ingredient | Anti-caries activity, whitening effects, halitosis control |
|  | Aqua | Solvents | Solvent |
|  | Sorbitol | Inactive - Sweeteners | Substitute for sugar used as a sweetening agent |
|  | Hydrated Silica | Whitening/plaque removal agents | Used as an abrasive, active in extrinsic stain and plaque removal |
|  | Sodium Lauryl Sulfate | Emulsifier | Thickening and foaming agent. Antimicrobial properties |
|  | Cellulose gum | Thickening agents | Thickening agent |
|  | Aroma | Inactive - Fragrances | Fragrance additive |
|  | Sodium Saccharin | Inactive - Sweeteners | Artificial sweetener |
|  | Sodium Hydroxide | Buffering agent | Buffering agent |
|  | Limonene | Inactive - Fragrances | Flavor and fragrance additive |
|  | CI 77891 | Artificial - Colorant + Whitening/plaque removal agents | Active in extrinsic stain removal |
| **Boots Kids Mild Mint Toothpaste Ages 3-5** | Sodium Fluoride 1450 ppm | Active ingredient | Anti-caries activity, whitening effects, halitosis control |
|  | Aqua | Solvents | Solvent |
|  | Sorbitol | Inactive - Sweeteners | Substitute for sugar used as a sweetening agent |
|  | Hydrated Silica | Whitening/plaque removal agents | Used as an abrasive, active in extrinsic stain and plaque removal |
|  | Sodium Lauryl Sulfate | Emulsifier | Thickening and foaming agent. Antimicrobial properties |
|  | Cellulose gum | Thickening agents | Thickening agent |
|  | Aroma | Inactive - Fragrances | Fragrance additive |
|  | Sodium Saccharin | Inactive - Sweeteners | Artificial sweetener |
|  | Sodium Hydroxide | Buffering agent | Buffering agent |
|  | Limonene | Inactive - Fragrances | Flavor and fragrance additive |
|  | CI 77891 | Artificial - Colorant + Whitening/plaque removal agents | Active in extrinsic stain removal |
| **R.O.C.S. Kids 3-7 Fruity Cone** | Aqua | Solvents | Solvent |
|  | Glycerin | Wetting agents | Used to prevent toothpaste from drying out |
|  | Silica | Abrasives | Abrasive agent |
|  | Xylitol (12%) | Whitening/plaque removal agents | Active in caries prevention + Effective against S. mutans |
|  | Cocamidopropyl Betaine | Surfactant | Thickening and foaming agent |
|  | Xanthan Gum | Thickening agents | Thickening and stabilizing agent |
|  | Calcium Glycerophosphate | Remineralization agents | Enhances the effects of F on enamel de- and re-mineralization processes. Reduced enamel erosion |
|  | Polysorbate-20 | Emulsifier | Wetting agent and stabilizer |
|  | Magnesium Chloride | Remineralization agents | Antimicrobial and anti-inflammatory properties |
|  | Flavor | Inactive - Flavorings | Flavoring agent |
|  | Sodium saccharin | Inactive - Sweeteners | Artificial sweetener |
|  | Methylparaben | Preserving agents | Preserving agent |
|  | Titanium Dioxide | Artificial - Colorant + Whitening/plaque removal agents | Active in extrinsic stain removal |
|  | Propyparaben | Preserving agents | Preserving agent |
|  | Limonene | Inactive - Fragrances | Flavor and fragrance additive |
| **R.O.C.S. Kids 3-7 Baeberry** | Aqua | Solvents | Solvent |
|  | Glycerin | Wetting agents | Used to prevent toothpaste from drying out |
|  | Silica | Abrasives | Abrasive agent |
|  | Xylitol (12%) | Whitening/plaque removal agents | Active in caries prevention + Effective against S. mutans |
|  | Cocamidopropyl Betaine | Surfactant | Thickening and foaming agent |
|  | Xanthan Gum | Thickening agents | Thickening and stabilizing agent |
|  | Calcium Glycerophosphate | Remineralization agents | Enhances the effects of F on enamel de- and re-mineralization processes. Reduced enamel erosion |
|  | Polysorbate-20 | Emulsifier | Wetting agent and stabilizer |
|  | Magnesium Chloride | Remineralization agents | Antimicrobial and anti-inflammatory properties |
|  | Sodium saccharin | Inactive - Sweeteners | Artificial sweetener |
|  | Methylparaben | Preserving agents | Preserving agent |
|  | Titanium Dioxide | Artificial - Colorant + Whitening/plaque removal agents | Active in extrinsic stain removal |
|  | Propyparaben | Preserving agents | Preserving agent |
|  | Limonene | Inactive - Fragrances | Flavor and fragrance additive |
|  | Aroma | Inactive - Fragrances | Fragrance additive |
| **R.O.C.S. Kids 3-7 Sweet Princess** | Aqua | Solvents | Solvent |
|  | Glycerin | Wetting agents | Used to prevent toothpaste from drying out |
|  | Silica | Abrasives | Abrasive agent |
|  | Xylitol (12%) | Whitening/plaque removal agents | Active in caries prevention + Effective against S. mutans |
|  | Cocamidopropyl Betaine | Surfactant | Thickening and foaming agent |
|  | Xanthan Gum | Thickening agents | Thickening and stabilizing agent |
|  | Calcium Glycerophosphate | Remineralization agents | Enhances the effects of F on enamel de- and re-mineralization processes. Reduced enamel erosion |
|  | Polysorbate-20 | Emulsifier | Wetting agent and stabilizer |
|  | Magnesium Chloride | Remineralization agents | Antimicrobial and anti-inflammatory properties |
|  | Sodium saccharin | Inactive - Sweeteners | Artificial sweetener |
|  | Methylparaben | Preserving agents | Preserving agent |
|  | Titanium Dioxide | Artificial - Colorant + Whitening/plaque removal agents | Active in extrinsic stain removal |
|  | Propyparaben | Preserving agents | Preserving agent |
|  | Limonene | Inactive - Fragrances | Flavor and fragrance additive |
|  | Aroma | Inactive - Fragrances | Fragrance additive |
|  | Benzyl Alcohol | Solvents | Solvent |
|  | Geraniol | Inactive - Fragrances | Flavor and fragrance additive |
|  | Citronellol | Inactive - Fragrances | Fragrance additive |
| **R.O.C.S. Kids 4-7 Summer Swirl** | Fluoride 500 ppm | Active ingredient | Anti-caries activity, whitening effects, halitosis control |
|  | Aqua | Solvents | Solvent |
|  | Glycerin | Wetting agents | Used to prevent toothpaste from drying out |
|  | Silica | Abrasives | Abrasive agent |
|  | Xylitol | Whitening/plaque removal agents | Active in caries prevention + Effective against S. mutans |
|  | Olaflur | Amine Fluoride | Active in caries prevention |
|  | Hydroxyethylcellulose | Thickening agents | Thickening agent |
|  | Polysorbate-20 | Emulsifier | Wetting agent and stabilizer |
|  | Aroma | Inactive - Fragrances | Fragrance additive |
|  | Cocamidopropyl Betaine | Surfactant | Thickening and foaming agent |
|  | Titanium Dioxide | Artificial - Colorant + Whitening/plaque removal agents | Active in extrinsic stain removal |
|  | Sodium Saccharin | Inactive - Sweeteners | Artificial sweetener |
|  | Methylparaben | Preserving agents | Preserving agent |
|  | Propyparaben | Preserving agents | Preserving agent |
|  | Potassium Hydroxide | pH stabilizer | Helps adjust the pH level |
|  | Benzyl Alcohol | Solvents | Solvent |
| **R.O.C.S. Kids 4-7 Bubble Gum** | Amino Fluoride 500 ppm | Amine Fluoride | Active in caries prevention |
|  | Aqua | Solvents | Solvent |
|  | Glycerin | Wetting agents | Used to prevent toothpaste from drying out |
|  | Silica | Abrasives | Abrasive agent |
|  | Xylitol 10% | Whitening/plaque removal agents | Active in caries prevention + Effective against S. mutans |
|  | Olaflur | Amine Fluoride | Active in caries prevention |
|  | Hydroxyethylcellulose | Thickening agents | Thickening agent |
|  | Polysorbate-20 | Emulsifier | Wetting agent and stabilizer |
|  | Flavor | Inactive - Flavorings | Flavoring agent |
|  | Cocamidopropyl Betaine | Surfactant | Thickening and foaming agent |
|  | Titanium Dioxide | Artificial - Colorant + Whitening/plaque removal agents | Active in extrinsic stain removal |
|  | Sodium Saccharin | Inactive - Sweeteners | Artificial sweetener |
|  | Methylparaben | Preserving agents | Preserving agent |
|  | Propyparaben | Preserving agents | Preserving agent |
|  | Potassium Hydroxide | pH stabilizer | Helps adjust the pH level |
|  | Benzyl Alcohol | Solvents | Solvent |
| **Oral-B Kids 3+ Years** | Sodium Fluoride 500 ppm | Active ingredient | Anti-caries activity, whitening effects, halitosis control |
|  | Sorbitol | Inactive - Sweeteners | Substitute for sugar used as a sweetening agent |
|  | Aqua | Solvents | Solvent |
|  | Hydrated Silica | Whitening/plaque removal agents | Used as an abrasive, active in extrinsic stain and plaque removal |
|  | Sodium Lauryl Sulfate | Emulsifier | Thickening and foaming agent. Antimicrobial properties |
|  | Trisodium Phosphate | Whitening/plaque removal agents | Not found |
|  | Aroma | Inactive - Fragrances | Fragrance additive |
|  | Cellulose gum | Thickening agents | Thickening agent |
|  | Sodium Phosphate | Emulsifier | Emulsifier |
|  | Sodium Saccharin | Inactive - Sweeteners | Artificial sweetener |
|  | Carbomer | Inactive - Emulsifier | Binder (thickener) |
|  | Limonene | Inactive - Fragrances | Flavor and fragrance additive |
|  | Polysorbate-80 | Emulsifier | Wetting agent and stabilizer |
|  | Sodium Hydroxide | Buffering agent | Buffering agent |
|  | CI 42090 | Inactive - Artificial colorant | Colorant |
| **Elgydium Kids 2/6 Years Old** | Nicomethanol Hydrofluoride 500 ppm | Active ingredient | Anticariogenic |
|  | Hydrogenated Starch Hydrolysate | Wetting agents | Used also as a sweetener (polyol (sugar alcohol)) |
|  | Water (Aqua) | Solvents | Solvent |
|  | Hydrated Silica | Whitening/plaque removal agents | Used as an abrasive, active in extrinsic stain and plaque removal |
|  | Cellulose gum | Thickening agents | Thickening agent |
|  | Flavor (Aroma) | Inactive - Fragrances | Fragrance additive |
|  | Nicotinyl Alcohol HF | Antiplaque | Antiplaque |
|  | PEG-12 | Surfactant | Act as penetration enhancers |
|  | PEG-12 Allyl Ether | Surfactant | Act as penetration enhancers |
|  | PEG-12 Dimethicone | Surfactant | Act as penetration enhancers |
|  | Sodium Benzoate | Preserving agents | Preserving agent |
|  | Sodium Lauroyl Sarcosinate | Emulsifier | Acts as am emulsifier to improve the toothpaste foaming and thickening properties |
|  | Sodium Propionate | Preserving agents | Preserving agent |
|  | Sodium Saccharin | Inactive - Sweeteners | Artificial sweetener |
|  | Tetrasodium EDTA | Chelating agent | Chelating agent |
|  | Yellow 5 (CI 19140) | Inactive - Artificial colorant | Colorant |
| **Pasta Del Capitano Baby Captain 3+ Strawberry Toothpaste** | Sodium Monofluorophosphate | Active ingredient | An active anticariogenic compound |
|  | Aqua | Solvents | Solvent |
|  | Sorbitol | Inactive - Sweeteners | Substitute for sugar used as a sweetening agent |
|  | Hydrated Silica | Whitening/plaque removal agents | Used as an abrasive, active in extrinsic stain and plaque removal |
|  | Xylitol | Whitening/plaque removal agents | Active in caries prevention + Effective against S. mutans |
|  | Polysorbate 60 | Emulsifier | Wetting agent and stabilizer |
|  | Zinc coco-sulfate | Surfactant | Cleansing agent |
|  | Aroma | Inactive - Fragrances | Fragrance additive |
|  | Cellulose gum | Thickening agents | Thickening agent |
|  | Betaine | Emulsifier | Moisturizer and foam stabilizer |
|  | Jojoba esters | Vegetable-based ingredients | Antimicrobial activity against S. aureus |
|  | Sodium Saccharin | Inactive - Sweeteners | Artificial sweetener |
|  | Sodium Carbonate | Whitening/plaque removal agents | Active in extrinsic stain and plaque removal |
|  | Calcium Glycerophosphate | Remineralization agents | Enhances the effects of F on enamel de- and re-mineralization processes. Reduced enamel erosion |
|  | Propylene Glycol | Surfactant | Act as penetration enhancers |
|  | Malva Sylvestris Leaf Extract | Vegetable-based ingredients | It’s a plant species native to Europe, northern Africa, and Asia. It promotes wound healing, and possessed antioxidant, anti-inflammatory, antibacterial, antiviral, and anticancer properties. It is also active against *S. mutans*, *S. aureus*, and *C. albicans* |
|  | CI 73360 - Red 30 | Inactive - Artificial colorant | Colorant |
|  | CI 77007 | Inactive - Artificial colorant | Colorant |
|  | CI 19140:1 | Inactive - Artificial colorant | Colorant |
|  | CI 45430 | Inactive - Artificial colorant | Colorant |
|  | CI 47005 | Inactive - Artificial colorant | Colorant |
| **Pasta Del Capitano Baby Captain +6 Strawberry Toothpaste** | Sodium Monofluorophosphate | Active ingredient | An active anticariogenic compound |
|  | Aqua | Solvents | Solvent |
|  | Sorbitol | Inactive - Sweeteners | Substitute for sugar used as a sweetening agent |
|  | Hydrated Silica | Whitening/plaque removal agents | Used as an abrasive, active in extrinsic stain and plaque removal |
|  | Xylitol | Whitening/plaque removal agents | Active in caries prevention + Effective against S. mutans |
|  | Polysorbate 60 | Emulsifier | Wetting agent and stabilizer |
|  | Zinc coco-sulfate | Surfactant | Cleansing agent |
|  | Aroma | Inactive - Fragrances | Fragrance additive |
|  | Cellulose gum | Thickening agents | Thickening agent |
|  | Betaine | Emulsifier | Moisturizer and foam stabilizer |
|  | Jojoba esters | Vegetable-based ingredients | Antimicrobial activity against *S. aureus* |
|  | Calcium Glycerophosphate | Remineralization agents | Enhances the effects of F on enamel de- and re-mineralization processes. Reduced enamel erosion |
|  | Tocopheryl Acetate | Preserving agents | Preserving agent. It is the stable form of Vitamin E. |
|  | Sodium Saccharin | Inactive - Sweeteners | Artificial sweetener |
|  | Sodium Carbonate | Whitening/plaque removal agents | Active in extrinsic stain and plaque removal |
|  | CI 42051 | Inactive - Artificial colorant | Colorant |
|  | CI 77007 | Inactive - Artificial colorant | Colorant |
| **Oral Face Kids Toothpaste Banana Flavor** | Sodium Fluoride 550 ppm | Active ingredient | Anti-caries activity, whitening effects, halitosis control |
|  | Sorbitol | Inactive - Sweeteners | Substitute for sugar used as a sweetening agent |
|  | Aqua | Solvents | Solvent |
|  | Hydrated Silica | Whitening/plaque removal agents | Used as an abrasive, active in extrinsic stain and plaque removal |
|  | PEG-32 | Surfactant | Act as penetration enhancers |
|  | Sodium Lauryl Sulfate | Emulsifier | Thickening and foaming agent. Antimicrobial properties |
|  | Aroma | Inactive - Fragrances | Fragrance additive |
|  | Glycerin | Wetting agents | Used to prevent toothpaste from drying out |
|  | Cellulose gum | Thickening agents | Thickening agent |
|  | Sodium Saccharin | Inactive - Sweeteners | Artificial sweetener |
|  | Tocopheryl Acetate | Preserving agents | Preserving agent. It is the stable form of Vitamin E |
|  | Sodium Sulfate | Preserving agents | Preserving agent |
|  | Calcium Gluconate | Active ingredient | Helps in remineralization |
|  | CI 19140 | Inactive - Artificial colorant | Colorant |
| **Oral Face Kids Toothpaste Apple & Kiwi Flavor** | Sodium Fluoride 550 ppm | Active ingredient | Anti-caries activity, whitening effects, halitosis control |
|  | Sorbitol | Inactive - Sweeteners | Substitute for sugar used as a sweetening agent |
|  | Aqua | Solvents | Solvent |
|  | Hydrated Silica | Whitening/plaque removal agents | Used as an abrasive, active in extrinsic stain and plaque removal |
|  | PEG-32 | Surfactant | Act as penetration enhancers |
|  | Sodium Lauryl Sulfate | Emulsifier | Thickening and foaming agent. Antimicrobial properties |
|  | Aroma | Inactive - Fragrances | Fragrance additive |
|  | Glycerin | Wetting agents | Used to prevent toothpaste from drying out |
|  | Cellulose gum | Thickening agents | Thickening agent |
|  | Sodium Saccharin | Inactive - Sweeteners | Artificial sweetener |
|  | Tocopheryl Acetate | Preserving agents | Preserving agent. It is the stable form of Vitamin E. |
|  | Sodium Sulfate | Preserving agents | Preserving agent |
|  | Calcium Gluconate | Active ingredient | Helps in remineralization |
|  | CI 19140 | Inactive - Artificial colorant | Colorant |
|  | CI 42090 | Inactive - Artificial colorant | Colorant |
| **Paw Patrol Kids Toothgel 3+** | Sodium Fluoride 450 ppm | Active ingredient | Anti-caries activity, whitening effects, halitosis control |
|  | Sorbitol | Inactive - Sweeteners | Substitute for sugar used as a sweetening agent |
|  | Aqua | Solvents | Solvent |
|  | Hydrated Silica | Whitening/plaque removal agents | Used as an abrasive, active in extrinsic stain and plaque removal |
|  | PEG-8 | Surfactant | Act as penetration enhancers |
|  | Sodium Lauryl Sulfate | Emulsifier | Thickening and foaming agent. Antimicrobial properties |
|  | Aroma | Inactive - Fragrances | Fragrance additive |
|  | Cellulose gum | Thickening agents | Thickening agent |
|  | Sodium Saccharin | Inactive - Sweeteners | Artificial sweetener |
|  | Panthenol | Humectant | Moisturizing, soothing, and anti-inflammatory properties |
|  | Sodium Benzoate | Preserving agents | Preserving agent |
|  | Calcium Glycerophosphate | Remineralization agents | Enhances the effects of F on enamel de- and re-mineralization processes. Reduced enamel erosion |
|  | CI 73360 | Inactive - Artificial colorant | Colorant |
| **Paw Patrol Skye Kids Toothgel 3+** | Sodium Fluoride 450 ppm | Active ingredient | Anti-caries activity, whitening effects, halitosis control |
|  | Sorbitol | Inactive - Sweeteners | Substitute for sugar used as a sweetening agent |
|  | Aqua | Solvents | Solvent |
|  | Hydrated Silica | Whitening/plaque removal agents | Used as an abrasive, active in extrinsic stain and plaque removal |
|  | PEG-8 | Surfactant | Act as penetration enhancers |
|  | Sodium Lauryl Sulfate | Emulsifier | Thickening and foaming agent. Antimicrobial properties |
|  | Aroma | Inactive - Fragrances | Fragrance additive |
|  | Cellulose gum | Thickening agents | Thickening agent |
|  | Sodium Saccharin | Inactive - Sweeteners | Artificial sweetener |
|  | Panthenol | Humectant | Moisturizing, soothing, and anti-inflammatory properties |
|  | Sodium Benzoate | Preserving agents | Preserving agent |
|  | Calcium Glycerophosphate | Remineralization agents | Enhances the effects of F on enamel de- and re-mineralization processes. Reduced enamel erosion |
|  | CI 73360 | Inactive - Artificial colorant | Colorant |
| **Ice Age Collision Course Toothpaste Tutti-frutti 2+ Years** | Sodium Fluoride 500 ppm | Active ingredient | Anti-caries activity, whitening effects, halitosis control |
|  | Sorbitol | Inactive - Sweeteners | Substitute for sugar used as a sweetening agent |
|  | Aqua | Solvents | Solvent |
|  | Hydrated Silica | Whitening/plaque removal agents | Used as an abrasive, active in extrinsic stain and plaque removal |
|  | Glycerin | Wetting agents | Used to prevent toothpaste from drying out |
|  | Aroma | Inactive - Fragrances | Fragrance additive |
|  | Cellulose gum | Thickening agents | Thickening agent |
|  | Sodium Lauroyl Sarcosinate | Emulsifier | Acts as am emulsifier to improve the toothpaste foaming and thickening properties |
|  | Sodium Saccharin | Inactive - Sweeteners | Artificial sweetener |
|  | Tocopheryl Acetate | Preserving agents | Preserving agent. It is the stable form of Vitamin E. |
|  | Sodium Benzoate | Preserving agents | Preserving agent |
|  | Sodium Gluconate | Chelating agent | Chelating agent |
|  | Calcium Gluconate | Active ingredient | Helps in remineralization |
| **Theodent Kids Chocolate Toothpaste** | Theobromine | Active ingredient | Antimicrobial and anti-plaque |
|  | Calcium Acetate | Remineralization agents | Remineralization agent |
|  | Sodium Hydrogen Phosphate | Emulsifier | Emulsifier |
|  | Purified Water | Solvents | Solvent |
|  | Hydrated Silica | Whitening/plaque removal agents | Used as an abrasive, active in extrinsic stain and plaque removal |
|  | Sorbitol | Inactive - Sweeteners | Substitute for sugar used as a sweetening agent |
|  | Xylitol | Whitening/plaque removal agents | Active in caries prevention + Effective against S. mutans |
|  | Glycerin | Wetting agents | Used to prevent toothpaste from drying out |
|  | Xanthan Gum | Thickening agents | Thickening and stabilizing agent |
|  | Citric Acid | Whitening/plaque removal agents | Active in dental plaque removal and whitening teeth |
|  | Sodium Benzoate | Preserving agents | Preserving agent |
|  | Titanium Dioxide | Artificial - Colorant + Whitening/plaque removal agents | Active in extrinsic stain removal |
|  | Stevia Extract | Inactive - Natural sweetener | Bio-sweetener. Anti-hypertensive, anti-obesity, anti-diabetic, antioxidant, anti-cancer, anti-inflammatory, and antimicrobial effects and improvement of kidney function |
|  | Sodium Bicarbonate | Whitening/plaque removal agents | Active in extrinsic stain and plaque removal |
|  | Sugar-free Chocolate and Vanilla Extract | Inactive - Flavorings | Flavoring agent |
| **BEN 10 Kids Toothgel 3+** | Sodium Fluoride 450 ppm | Active ingredient | Anti-caries activity, whitening effects, halitosis control |
|  | Aqua | Solvents | Solvent |
|  | Sorbitol | Inactive - Sweeteners | Substitute for sugar used as a sweetening agent |
|  | Hydrated Silica | Whitening/plaque removal agents | Used as an abrasive, active in extrinsic stain and plaque removal |
|  | PEG-8 | Surfactant | Act as penetration enhancers |
|  | Sodium Lauryl Sulfate | Emulsifier | Thickening and foaming agent. Antimicrobial properties |
|  | Sodium Saccharin | Inactive - Sweeteners | Artificial sweetener |
|  | Aroma | Inactive - Fragrances | Fragrance additive |
|  | Cellulose gum | Thickening agents | Thickening agent |
|  | Panthenol | Humectant | Moisturizing, soothing, and anti-inflammatory properties |
|  | Sodium Benzoate | Preserving agents | Preserving agent |
|  | Calcium Glycerophosphate | Remineralization agents | Enhances the effects of F on enamel de- and re-mineralization processes. Reduced enamel erosion |
|  | CI 73360 | Inactive - Artificial colorant | Colorant |
| **G.U.M Junior 7+** | Sorbitol | Inactive - Sweeteners | Substitute for sugar used as a sweetening agent |
|  | Aqua | Solvents | Solvent |
|  | Hydrated Silica | Whitening/plaque removal agents | Used as an abrasive, active in extrinsic stain and plaque removal |
|  | Isomalt | Inactive - Sweeteners | Sugar alcohol |
|  | Cellulose gum | Thickening agents | Thickening agent |
|  | Aroma | Inactive - Fragrances | Fragrance additive |
|  | Disodium Phosphate | Preservative and a stabilizer | Stabilises dental products and controls acidity |
|  | Lauryl Glucoside | Surfactant | Foaming agent |
|  | Sodium Saccharin | Inactive - Sweeteners | Artificial sweetener |
|  | Sodium Benzoate | Preserving agents | Preserving agent |
|  | Sodium Fluoride | Active ingredient | Anti-caries activity, whitening effects, halitosis control |
|  | Sodium Phosphate | Emulsifier | Emulsifier |
|  | CI 73360 | Inactive - Artificial colorant | Colorant |
|  | Mica | Whitening/plaque removal agents | Mild abrasive |
|  | CI 47005 | Inactive - Artificial colorant | Colorant |
|  | Titanium Dioxide (CI 77891) (E171) | Artificial - Colorant + Whitening/plaque removal agents | Active in extrinsic stain removal |
|  | CI 16035 | Inactive - Artificial colorant | Colorant |
| **Sensodyne Pronamel for Children 6+ Years** | Aqua | Solvents | Solvent |
|  | Sorbitol | Inactive - Sweeteners | Substitute for sugar used as a sweetening agent |
|  | Hydrated Silica | Whitening/plaque removal agents | Used as an abrasive, active in extrinsic stain and plaque removal |
|  | Glycerin | Wetting agents | Used to prevent toothpaste from drying out |
|  | PEG-6 | Surfactant | Act as penetration enhancers |
|  | Cocamidopropyl Betaine | Surfactant | Thickening and foaming agent |
|  | Xanthan Gum | Thickening agents | Thickening and stabilizing agent |
|  | Aroma | Inactive - Fragrances | Fragrance additive |
|  | Sodium Saccharin | Inactive - Sweeteners | Artificial sweetener |
|  | Sucralose | Inactive - Sweeteners | Artificial sweetener. Commonly known as "Splenda" and is used in many food products |
|  | Titanium Dioxide | Artificial - Colorant + Whitening/plaque removal agents | Active in extrinsic stain removal |
|  | Sodium Hydroxide | Buffering agent | Buffering agent |
|  | Limonene | Inactive - Fragrances | Flavor and fragrance additive |
|  | Sodium Fluoride 1450 ppm | Active ingredient | Anti-caries activity, whitening effects, halitosis control |
| **Firefly Avengers** | Sorbitol | Inactive - Sweeteners | Substitute for sugar used as a sweetening agent |
|  | Hydrated Silica | Whitening/plaque removal agents | Used as an abrasive, active in extrinsic stain and plaque removal |
|  | Aqua | Solvents | Solvent |
|  | Propylene Glycol | Surfactant | Act as penetration enhancers |
|  | Sodium Lauryl Sulfate | Emulsifier | Thickening and foaming agent. Antimicrobial properties |
|  | Flavor | Inactive - Flavorings | Functions as a flavoring agent |
|  | Cellulose Gum | Thickening agents | Thickening agent |
|  | Sodium Monofluorophosphate 1050 ppm | Active ingredient | An active anticariogenic compound |
|  | Sodium Saccharin | Inactive - Sweeteners | Artificial sweetener |
|  | Sodium Benzoate | Preserving agents | Preserving agent |
|  | Cl 42090 | Inactive - Artificial colorant | Colorant |
| **Firefly Spiderman** | Sorbitol | Inactive - Sweeteners | Substitute for sugar used as a sweetening agent |
|  | Silica | Abrasives | Abrasive agent |
|  | Aqua | Solvents | Solvent |
|  | Propylene Glycol | Surfactant | Act as penetration enhancers |
|  | Sodium Lauryl Sulfate | Emulsifier | Thickening and foaming agent. Antimicrobial properties |
|  | Aroma | Inactive - Fragrances | Fragrance additive |
|  | Cellulose Gum | Thickening agents | Thickening agent |
|  | Sodium Monofluorophosphate 1050 ppm | Active ingredient | An active anticariogenic compound |
|  | Sodium Saccharin | Inactive - Sweeteners | Artificial sweetener |
|  | Sodium Benzoate | Preserving agents | Preserving agent |
|  | Eugenol | Inactive - Fragrances | Flavor and fragrance additive |
|  | Cl 42090 | Inactive - Artificial colorant | Colorant |
| **Oradentex Kids** | Sorbitol | Inactive - Sweeteners | Substitute for sugar used as a sweetening agent |
|  | Aqua | Solvents | Solvent |
|  | Hydrated Silica | Whitening/plaque removal agents | Used as an abrasive, active in extrinsic stain and plaque removal |
|  | PEG-8 | Surfactant | Act as penetration enhancers |
|  | Sodium Lauryl Sulfate | Emulsifier | Thickening and foaming agent. Antimicrobial properties |
|  | Aroma | Inactive - Fragrances | Fragrance additive |
|  | Xanthan Gum | Thickening agents | Thickening and stabilizing agent |
|  | Sodium Saccharin | Inactive - Sweeteners | Artificial sweetener |
|  | Sodium Benzoate | Preserving agents | Preserving agent |
|  | Calcium Glycerophosphate | Remineralization agents | Enhances the effects of F on enamel de- and re-mineralization processes. Reduced enamel erosion |
|  | Sodium Fluoride 450 ppm | Active ingredient | Anti-caries activity, whitening effects, halitosis control |
|  | Tocopheryl Acetate | Preserving agents | Preserving agent. It is the stable form of Vitamin E. |
|  | Cl 73360 | Inactive - Artificial colorant | Colorant |
| **Nickelodeon Air-Val Dora Explorer Kids Tooth Paste** | Aqua | Solvents | Solvent |
|  | Sorbitol | Inactive - Sweeteners | Substitute for sugar used as a sweetening agent |
|  | Hydrated Silica | Whitening/plaque removal agents | Used as an abrasive, active in extrinsic stain and plaque removal |
|  | PEG-8 | Surfactant | Act as penetration enhancers |
|  | Sodium Lauryl Sulfate | Emulsifier | Thickening and foaming agent. Antimicrobial properties |
|  | Sodium Saccharin | Inactive - Sweeteners | Artificial sweetener |
|  | Aroma | Inactive - Fragrances | Fragrance additive |
|  | Cellulose Gum | Thickening agents | Thickening agent |
|  | Panthenol | Humectant | Moisturizing, soothing, and anti-inflammatory properties |
|  | Sodium Benzoate | Preserving agents | Preserving agent |
|  | Calcium Glycerophosphate | Remineralization agents | Enhances the effects of F on enamel de- and re-mineralization processes. Reduced enamel erosion |
|  | Sodium Fluoride 450 ppm | Active ingredient | Anti-caries activity, whitening effects, halitosis control |
|  | Cl 73360 | Inactive - Artificial colorant | Colorant |
| **Oral-B Junior 6+ Star Wars** | Sodium Fluoride | Active ingredient | Anti-caries activity, whitening effects, halitosis control |
|  | Water | Solvents | Solvent |
|  | Hydrated Silica | Whitening/plaque removal agents | Used as an abrasive, active in extrinsic stain and plaque removal |
|  | Sorbitol | Inactive - Sweeteners | Substitute for sugar used as a sweetening agent |
|  | Sodium Lauryl Sulfate | Emulsifier | Thickening and foaming agent. Antimicrobial properties |
|  | Cellulose Gum | Thickening agents | Thickening agent |
|  | Flavor | Inactive - Flavorings | Flavoring agent |
|  | Trisodium Phosphate | Whitening/plaque removal agents | Not found |
|  | Carbomer | Inactive - Emulsifier | Binder (thickener) |
|  | Sodium Saccharin | Inactive - Sweeteners | Artificial sweetener |
|  | Polysorbate-80 | Emulsifier | Wetting agent and stabilizer |
|  | Cl 77891 | Artificial - Colorant + Whitening/plaque removal agents | Active in extrinsic stain removal |
|  | CI 74260 | Inactive - Artificial colorant | Colorant |
| **Biorepair Junior 6-12 Toothpaste** | Aqua | Solvents | Solvent |
|  | Glycerin | Wetting agents | Used to prevent toothpaste from drying out |
|  | Zinc Hydroxyapatite | Microrepair |  |
|  | Sorbitol | Inactive - Sweeteners | Substitute for sugar used as a sweetening agent |
|  | PEG-32 | Surfactant | Act as penetration enhancers |
|  | Silica | Abrasives | Abrasive agent |
|  | Sodium Myristoyt Sarcosinate | Emulsifier | pH buffering agent |
|  | Sodium Methyl Cocoyl Taurate | Surfactant | Foaimng agent |
|  | Cellulose Gum | Thickening agents | Thickening agent |
|  | Aroma | Inactive - Fragrances | Fragrance additive |
|  | Tocopheryl Acetate | Preserving agents | Preserving agent. It is the stable form of Vitamin E. |
|  | Sodium Saccharin | Inactive - Sweeteners | Artificial sweetener |
|  | Citric Acid | Whitening/plaque removal agents | Active in dental plaque removal and whitening teeth |
|  | Sodium Benzoate | Preserving agents | Preserving agent |
|  | Phenoxyethanol | Preserving agents | Preserving agent |
|  | Benzyl Alcohol | Solvents | Solvent |
