# Supplementary material for: Labeled ingredient composition and literature-reported hazard signals in children's toothpastes available through selected UAE retail sources
Source: Front Oral Health. 2026 Aug 26;7:1932715. doi: 10.3389/froh.2026.1932715 (PMC13553250; doi:10.3389/froh.2026.1932715)
Supplement: Supplementary file 2 [file Table2.docx]

**APPENDIX 2 Children’s Toothpastes Labeled as Natural, Herbal, and Organic in the UAE Market with a Description of their Ingredients**

| **Toothpaste** | **Ingredients** | **Type of ingredient** | **Purpose** |
| --- | --- | --- | --- |
| **Colgate 0-2 Years Natural Fruity Flavor** | **Propylene Glycol** | Surfactant | Act as penetration enhancers |
|  | **Glycerin** | Wetting agents | Used to prevent toothpaste from drying out |
|  | **Hydrated Silica** | Whitening/plaque removal agents | Used as an abrasive, active in extrinsic stain and plaque removal |
|  | **Sorbitol** | Inactive - Sweeteners | Substitute for sugar used as a sweetening agent |
|  | Aqua | Solvents | Solvent |
|  | **Xylitol** | Whitening/plaque removal agents | Active in caries prevention + Effective against S. mutans |
|  | **Polaxamer 407** | Surfactant | Surfactant and foaming agent |
|  | **Cellulose Gum** | Thickening agents | Thickening agent |
|  | **Citric Acid** | Whitening/plaque removal agents | Active in dental plaque removal and whitening teeth |
|  | **Aroma** | Inactive - Fragrances | Fragrance additive |
| **Colgate 3-5 Years Mint Flavor** | **Calcium Carbonate** | Abrasives | Used as an abrasive, which helps remove extrinsic stains and plaque |
|  | Aqua | Solvents | Solvent |
|  | **Glycerin** | Wetting agents | Used to prevent toothpaste from drying out |
|  | Arginine | Anticariogenic. It’s a prebiotic-based organic compound. It is a naturally occurring amino acid | It is a natural amino acid that shows promise as an ecological approach to managing dental caries. It can neutralize the acids that cause tooth decay and increase the production of beneficial bacteria in the mouth, such as *Streptococcus salivarius,* which can help to prevent the growth of harmful bacteria that can cause dental caries. Effective against *S. mutans* |
|  | **Cellulose Gum** | Thickening agents | Thickening agent |
|  | **Sodium Lauryl Sulfate** | Emulsifier | Thickening and foaming agent. Antimicrobial properties |
|  | **Benzyl Alcohol** | Solvents | Solvent |
|  | **Tetrasodium Pyrophosphate** | Chelating agent | Anti-calculus and whitening agent |
|  | **Sodium Bicarbonate** | Whitening/plaque removal agents | Active in extrinsic stain and plaque removal |
|  | Stevia Rebaudiana Extract | Inactive - Natural sweetener | Bio-sweetener. Anti-hypertensive, anti-obesity, anti-diabetic, antioxidant, anti-cancer, anti-inflammatory, and antimicrobial effects and improvement of kidney function |
|  | **Aroma** | Inactive - Fragrances | Fragrance additive |
|  | **Phosphoric Acid** | Buffering agents, corrosion inhibitors, chelating agents, and pH adjusters | pH adjuster, corrosion inhibitor, chelating agent, and buffering agent |
|  | Rebaudioside A | Inactive - Artificial sweetener | A highly purified form of stevia. Bio-sweetener. Anti-hypertensive, anti-obesity, anti-diabetic, antioxidant, anti-cancer, anti-inflammatory, and antimicrobial effects and improvement of kidney function |
|  | **Maltodextrin** | Thickening agents | Thickening and bulking agent |
|  | **Limonene** | Inactive - Fragrances | Flavor and fragrance additive |
| **Colgate 6-9 Years Strawbery Mint Flavor** | **Sorbitol** | Inactive - Sweeteners | Substitute for sugar used as a sweetening agent |
|  | Aqua | Solvents | Solvent |
|  | **Hydrated Silica** | Whitening/plaque removal agents | Used as an abrasive, active in extrinsic stain and plaque removal |
|  | **Xylitol** | Whitening/plaque removal agents | Active in caries prevention + Effective against S. mutans |
|  | **PEG-12** | Surfactant | Act as penetration enhancers |
|  | **Cellulose Gum** | Thickening agents | Thickening agent |
|  | **Benzyl Alcohol** | Solvents | Solvent |
|  | **Sodium Lauryl Sulfate** | Emulsifier | Thickening and foaming agent. Antimicrobial properties |
|  | **Aroma** | Inactive - Fragrances | Fragrance additive |
|  | **Sodium Fluoride 1000 ppm** | Active ingredient | Anti-caries activity, whitening effects, halitosis control |
|  | **Linalool** | Inactive - Fragrances | Flavor and fragrance additive. Has a terpene fragrance with a floral-woody smell and a hint of citrus found in over 200 natural oils |
| **Curaprox Kids Strawberry 0+** | Aqua | Solvents | Solvent |
|  | **Glycerin** | Wetting agents | Used to prevent toothpaste from drying out |
|  | **Hydrated Silica** | Whitening/plaque removal agents | Used as an abrasive, active in extrinsic stain and plaque removal |
|  | **Sorbitol** | Inactive - Sweeteners | Substitute for sugar used as a sweetening agent |
|  | Xylitol | Whitening/plaque removal agents | Active in caries prevention + Effective against S. mutans |
|  | **Xanthan Gum** | Thickening agents | Thickening and stabilizing agent |
|  | Aroma | Inactive - Fragrances | Fragrance additive |
|  | **Lauryl Glucoside** | Surfactant | Foaming agent |
|  | **Cocamidopropyl Betaine** | Surfactant | Thickening and foaming agent |
|  | **Sucralose** | Inactive - Sweeteners | Artificial sweetener. Commonly known as "Splenda" and is used in many food products |
|  | Amyloglucosidase | Enzyme | Can boost the natural salivary defences and have an anti-caries effect. Can reduce plaque accumelation and gingivitis |
|  | Glucose Oxidase | Enzyme | Anti-caries and anti-plaque effect |
|  | Polylysine | Preseeving agents | Preservig agent and antimicrobial activity |
|  | **Propyl Gallate** | Preseeving agents | Preserving agent by acting as an antioxidant |
|  | Sodium Citrate | Desensetizers | Help relieve sensitivity (pH buffer) |
|  | **Citric Acid** | Whitening/plaque removal agents | Active in dental plaque removal and whitening teeth |
|  | **Sodium Hydroxide** | Buffering agent | Buffering agent |
| **Curaprox Kids Strawberry 2+** | Sodium Monofluorophosphate 950 ppm | Active ingredient | An active anticariogenic compound |
|  | Aqua | Solvents | Solvent |
|  | Glycerin | Wetting agents | Used to prevent toothpaste from drying out |
|  | Hydrated Silica | Whitening/plaque removal agents | Used as an abrasive, active in extrinsic stain and plaque removal |
|  | Sorbitol | Inactive - Sweeteners | Substitute for sugar used as a sweetening agent |
|  | Xylitol | Whitening/plaque removal agents | Active in caries prevention + Effective against S. mutans |
|  | Xanthan Gum | Thickening agents | Thickening and stabilizing agent |
|  | Aroma | Inactive - Fragrances | Fragrance additive |
|  | Lauryl Glucoside | Surfactant | Foaming agent |
|  | Cocamidopropyl Betaine | Surfactant | Thickening and foaming agent |
|  | Sucralose | Inactive - Sweeteners | Artificial sweetener. Commonly known as "Splenda" and is used in many food products |
|  | Amyloglucosidase | Enzyme | Can boost the natural salivary defences and have an anti-caries effect. Can reduce plaque accumelation and gingivitis |
|  | Glucose Oxidase | Enzyme | Anti-caries and anti-plaque effect |
|  | Polylysine | Preseeving agents | Preservig agent and antimicrobial activity |
|  | Propyl Gallate | Preseeving agents | Preserving agent by acting as an antioxidant |
|  | Sodium Citrate | Desensetizers | Help relieve sensitivity (pH buffer) |
|  | Citric Acid | Whitening/plaque removal agents | Active in dental plaque removal and whitening teeth |
|  | Sodium Hydroxide | Buffering agent | Buffering agent |
| **Curaprox Kids Sweet Mint From 6 Years** | Sodium Monofluorophosphate 1450 ppm | Active ingredient | An active anticariogenic compound |
|  | Aqua | Solvents | Solvent |
|  | Glycerin | Wetting agents | Used to prevent toothpaste from drying out |
|  | Hydrated Silica | Whitening/plaque removal agents | Used as an abrasive, active in extrinsic stain and plaque removal |
|  | Sorbitol | Inactive - Sweeteners | Substitute for sugar used as a sweetening agent |
|  | Xylitol | Whitening/plaque removal agents | Active in caries prevention + Effective against S. mutans |
|  | Xanthan Gum | Thickening agents | Thickening and stabilizing agent |
|  | Aroma | Inactive - Fragrances | Fragrance additive |
|  | Lauryl Glucoside | Surfactant | Foaming agent |
|  | Cocamidopropyl Betaine | Surfactant | Thickening and foaming agent |
|  | Sucralose | Inactive - Sweeteners | Artificial sweetener. Commonly known as "Splenda" and is used in many food products |
|  | Amyloglucosidase | Enzyme | Can boost the natural salivary defences and have an anti-caries effect. Can reduce plaque accumelation and gingivitis |
|  | Glucose Oxidase | Enzyme | Anti-caries and anti-plaque effect |
|  | Polylysine | Preseeving agents | Preservig agent and antimicrobial activity |
|  | Propyl Gallate | Preseeving agents | Preserving agent by acting as an antioxidant |
|  | Sodium Citrate | Desensetizers | Help relieve sensitivity (pH buffer) |
|  | Citric Acid | Whitening/plaque removal agents | Active in dental plaque removal and whitening teeth |
|  | Sodium Hydroxide | Buffering agent | Buffering agent |
|  | Limonene | Inactive - Fragrances | Flavor and fragrance additive |
| **Curaprox Kids Watermelon 6+** | Sodium Monofluorophosphate 1450 ppm | Active ingredient | An active anticariogenic compound |
|  | Aqua | Solvents | Solvent |
|  | Glycerin | Wetting agents | Used to prevent toothpaste from drying out |
|  | Hydrated Silica | Whitening/plaque removal agents | Used as an abrasive, active in extrinsic stain and plaque removal |
|  | Sorbitol | Inactive - Sweeteners | Substitute for sugar used as a sweetening agent |
|  | Xylitol | Whitening/plaque removal agents | Active in caries prevention + Effective against S. mutans |
|  | Xanthan Gum | Thickening agents | Thickening and stabilizing agent |
|  | Aroma | Inactive - Fragrances | Fragrance additive |
|  | Lauryl Glucoside | Surfactant | Foaming agent |
|  | Cocamidopropyl Betaine | Surfactant | Thickening and foaming agent |
|  | Sucralose | Inactive - Sweeteners | Artificial sweetener. Commonly known as "Splenda" and is used in many food products |
|  | Amyloglucosidase | Enzyme | Can boost the natural salivary defences and have an anti-caries effect. Can reduce plaque accumelation and gingivitis |
|  | Glucose Oxidase | Enzyme | Anti-caries and anti-plaque effect |
|  | Polylysine | Preseeving agents | Preservig agent and antimicrobial activity |
|  | Propyl Gallate | Preseeving agents | Preserving agent by acting as an antioxidant |
|  | Sodium Citrate | Desensetizers | Help relieve sensitivity (pH buffer) |
|  | Citric Acid | Whitening/plaque removal agents | Active in dental plaque removal and whitening teeth |
|  | Sodium Hydroxide | Buffering agent | Buffering agent |
|  | Limonene | Inactive - Fragrances | Flavor and fragrance additive |
|  | Hydroxycitronellal | Citronella - Inactive - Fragrances | Flavor and fragrance additive |
|  | Linalool | Inactive - Fragrances | Flavor and fragrance additive. Has a terpene fragrance with a floral-woody smell and a hint of citrus found in over 200 natural oils |
|  | Benzyl Alcohol | Solvents | Solvent |
| **R.O.C.S baby 0-3 years** | Aqua | Solvents | Solvent |
|  | Glycerin | Wetting agents | Used to prevent toothpaste from drying out |
|  | Xylitol (10%) | Whitening/plaque removal agents | Active in caries prevention + Effective against S. mutans |
|  | Silica | Abrasives | Abrasive agent |
|  | Chamomilla Recutita (Matricaria) Flower Extract | Vegetable-based ingredients | It is an annual herb that grows in Southeast Europe and adjacent Asian countries, has been used for medicinal purposes since ancient times. The Egyptians considered it a sacred gift from the God 'Sun'. German chamomile is known as a "star" herb due to its aromatic and pharmacological properties, which include anti-inflammatory, antiulcerogenic, antimicrobial, antiseptic, antispasmodic, sedative, immunomodulatory, and wound-healing effects |
|  | Potassium (Sodium) Alginate | Thickening agents | Sodium alginate is a salt made from brown algae that is commonly used as a thickening agent in toothpastes |
|  | Lonicera Caprifolium Extract | Natural preservative | Natual preservative. Has anti-bacterial, anti-viral, and anti-inflammatory properties |
|  | Lonicera Japonica Extract | Natural preservative | Natual preservative. Has anti-bacterial, anti-viral, and anti-inflammatory properties |
|  | Sodium Benzoate | Preserving agents | Preserving agent |
|  | Xanthan Gum | Thickening agents | Thickening and stabilizing agent |
| **R.O.C.S baby 0-3 PRO** | Aqua | Solvents | Solvent |
|  | Glycerin* | Wetting agents | Used to prevent toothpaste from drying out |
|  | Dicalcium Phosphate Dihydrate* | Remineralization agents | Help enhance the remineralizing effect of fluoride |
|  | Xylitol (10%)* | Whitening/plaque removal agents | Active in caries prevention + Effective against S. mutans |
|  | Silica* | Abrasives | Abrasive agent |
|  | Calcium Glycerophosphate | Remineralization agents | Enhances the effects of F on enamel de- and re-mineralization processes. Reduced enamel erosion |
|  | Tillia Cordata Flower Extract* | Natural flavoring agent | It is a natural extract from the Linden tree's flowers, used in cosmetics for its antibacterial and anti-inflammatory properties, and as a natural flavoring agent. It contains flavonoids and antioxidants like tiliroside, quercetin, and kaempferol, which eliminate free radicals and prevent inflammation and oxidative damage. Kaempferol has shown potential in cancer-fighting studies |
|  | Xanthan Gum* | Thickening agents | Thickening and stabilizing agent |
|  | Lonicera Caprifolium Extract* | Natural preservative | Natual preservative. Has anti-bacterial, anti-viral, and anti-inflammatory properties |
|  | Lonicera Japonica Extract* | Natural preservative | Natual preservative. Has anti-bacterial, anti-viral, and anti-inflammatory properties |
|  | Sodium Benzoate* | Preserving agents | Preserving agent |
|  | Magnesium Chloride | Remineralization agents | Antimicrobial and anti-inflammatory properties |
| **R.O.C.S Junior 6-12 Berry mix** | Aqua | Solvents | Solvent |
|  | Glycerin | Wetting agents | Used to prevent toothpaste from drying out |
|  | Silica | Abrasives | Abrasive agent |
|  | Xylitol (12%) | Whitening/plaque removal agents | Active in caries prevention + Effective against S. mutans |
|  | Cocamidopropyl Betaine | Surfactant | Thickening and foaming agent |
|  | Sodium Lauroyl Sarcosinate | Emulsifier | Acts as am emulsifier to improve the toothpaste foaming and thickening properties |
|  | Populus Tremuloides Bark Extract | Natural organic preservative | Natural and organic preservative that contains a high amount of salicylates, which are known to have strong antimicrobial properties |
|  | Xanthan Gum | Thickening agents | Thickening and stabilizing agent |
|  | Aroma | Inactive - Fragrances | Fragrance additive |
|  | Calcium Glycerophosphate | Remineralization agents | Enhances the effects of F on enamel de- and re-mineralization processes. Reduced enamel erosion |
|  | Hydroxyacetophenone | Preservative | Preservative enhancer, antioxidant, and calming agent |
|  | Sodium Benzoate | Preserving agents | Preserving agent |
|  | Potassium Sorbate | Preserving agents | Preserving agent |
|  | Sodium Saccharin | Inactive - Sweeteners | Artificial sweetener |
|  | Magnesium Chloride | Remineralization agents | Antimicrobial and anti-inflammatory properties |
|  | O-cymen-5-ol | Preseeving agents | It is utilized in toothpaste as an antimicrobial agent and a preservative |
|  | Potassium Hydroxide | pH stabilizer | Helps adjust the pH level |
| **SPLAT kids natural toothpaste for kids 2-6 years Fruit Ice-cream flavor** | Aqua* | Solvents | Solvent |
|  | Hydrogenated Starch Hydrolysate* | Wetting agents | Used also as a sweetener (polyol (sugar alcohol)) |
|  | Glycerin* | Wetting agents | Used to prevent toothpaste from drying out |
|  | Hydrated Silica* | Whitening/plaque removal agents | Used as an abrasive, active in extrinsic stain and plaque removal |
|  | Calcium Hydroxyapatite* | Remineralization agents | Has been shown to improve the hardness of the enamel surface and contribute to remineralization, which is similar to the effects of fluoride. As a result, it is an effective substitute for fluoride and can help prevent fluorosis |
|  | Cellulose Gum* | Thickening agents | Thickening agent |
|  | Aroma* | Inactive - Fragrances | Fragrance additive |
|  | Sodium Benzoate | Preserving agents | Preserving agent |
|  | Cocamidopropyl Betaine* | Surfactant | Thickening and foaming agent |
|  | Xanthan Gum* | Thickening agents | Thickening and stabilizing agent |
|  | Potassium Sorbate | Preserving agents | Preserving agent |
|  | Lonicera Japonica Flower Extract* | Natural preservative | Natual preservative. Has anti-bacterial, anti-viral, and anti-inflammatory properties |
|  | Maltitol* | Inactive - Artificial sweetener - a sugar alcohol | Artificial sweetener |
|  | Sodium Chloride* | Abrasives | Abrasive agent |
|  | Lonicera Caprifolium Flower Extract* | Natural preservative | Natual preservative. Has anti-bacterial, anti-viral, and anti-inflammatory properties |
|  | Vitis Vinifera Seed Extract* | Vegetable-based ingredients | Caries prevention. Antioxidant, antibacterial, and anti-inflammatory properties |
|  | Pentylene Glycol* | Humectant | A preservative, solvent, and humectant |
|  | Arginine* | Anticariogenic. It’s a prebiotic-based organic compound. It is a naturally occurring amino acid | It is a natural amino acid that shows promise as an ecological approach to managing dental caries. It can neutralize the acids that cause tooth decay and increase the production of beneficial bacteria in the mouth, such as *Streptococcus salivarius,* which can help to prevent the growth of harmful bacteria that can cause dental caries. Effective against *S. mutans* |
|  | Potassium Thiocyanate | Humectant | Preserving agent, stabilizer, and antibacterial |
|  | Aloe Barbadensis Leaf Extract* | Vegetable-based ingredients | Emollient and antimicrobial agent; used to treat aphthous ulcers and to reduce the incidence of alveolar osteitis after third molar extraction surgeries; a mouth rinse containing A. vera was found to reduce gingival inflammation and gingival bleeding and was more effective than Listerine® in reducing the count of aerobic, microaerophilic and anaerobic bacteria |
|  | Lactoferrin* | Protein | Has been found to possess anti-inflammatory, anti-microbial, and anti-viral properties. Effective against *S. mutans* |
|  | Glycyrrhiza Glabra Root Extract* | .Vegetable-based ingredients. It’s a sweet and soothing herb originating from the Glycyrrhiza glabra plant, has been widely used as a sweetener and flavoring agent in food and medicine production. | Possesses anti-inflammatory, anti-oxidant, and anti-microbial properties, effective in treating dental caries, gingivitis, periodontitis, aphthous ulcers, and oral cancer. Reduces the count of *S. mutans* |
|  | Citric Acid* | Whitening/plaque removal agents | Active in dental plaque removal and whitening teeth |
|  | Helianthus Annuus Seed Oil* | Emollients | It is potent antioxidant that can help maintain healthy cholesterol levels and protect against cardiovascular diseases by promoting good blood flow. It also contains linoleic acid, vitamin E, and vitamin A, all of which are beneficial for health |
|  | Lactoperoxidase* | Enzyme | Has anticariogenic, antibacterial, antifungal, and antiviral properties. Effective against *S. mutans* |
|  | Zea Mays Starch* | Emulsifiers and Abrasives | Contains a large amount of polymeric carbohydrates and is typically produced from the peeled seeds of the corn plant (Zea mays). It is utilized in toothpastes as an emulsifier and abrasive agent |
|  | Glucose Pentaacetate | Emulsifiers | Emulsifier, stabilizer, and fragrance |
|  | Glucose Oxidase* | Enzyme | Anti-caries and anti-plaque effect |
|  | Xanthophylls* | Inactive - Natural colorant | Natural colorant |
|  | Ascorbyl Palmitate | Preser | Preservative and antioxidant |
|  | Tocopherol | Antioxidant | Antioxidant |
| **Jordan Green Clean Junior 6+ years** | Aqua | Solvents | Solvent |
|  | Sorbitol | Inactive - Sweeteners | Substitute for sugar used as a sweetening agent |
|  | Hydrated Silica | Whitening/plaque removal agents | Used as an abrasive, active in extrinsic stain and plaque removal |
|  | Xylitol | Whitening/plaque removal agents | Active in caries prevention + Effective against S. mutans |
|  | Xanthan Gum | Thickening agents | Thickening and stabilizing agent |
|  | Glycolipids | Natural surfactant | Natural surfactant |
|  | Stevia Rebaudiana Extract | Inactive - Natural sweetener | Bio-sweetener. Anti-hypertensive, anti-obesity, anti-diabetic, antioxidant, anti-cancer, anti-inflammatory, and antimicrobial effects and improvement of kidney function |
|  | Chamomilla Recutita (Matricaria) Flower Extract | Vegetable-based ingredients | It is an annual herb that grows in Southeast Europe and adjacent Asian countries, has been used for medicinal purposes since ancient times. The Egyptians considered it a sacred gift from the God 'Sun'. German chamomile is known as a "star" herb due to its aromatic and pharmacological properties, which include anti-inflammatory, antiulcerogenic, antimicrobial, antiseptic, antispasmodic, sedative, immunomodulatory, and wound-healing effects |
|  | Glycerin | Wetting agents | Used to prevent toothpaste from drying out |
|  | **Sodium Fluoride (1450 ppm)** | Active ingredient | Anti-caries activity, whitening effects, halitosis control |
|  | Potassium Sorbate | Preserving agents | Preserving agent |
|  | Citric Acid | Whitening/plaque removal agents | Active in dental plaque removal and whitening teeth |
|  | Sorbic Acid | Preserving agents | Preserving agent |
|  | Aroma | Inactive - Fragrances | Fragrance additive |
| **AloeDent Chidren's Strawberry Flavor** | Glycerin (veg origin) | Wetting agents | Used to prevent toothpaste from drying out |
|  | Sorbitol | Inactive - Sweeteners | Substitute for sugar used as a sweetening agent |
|  | Hydrated Silica | Whitening/plaque removal agents | Used as an abrasive, active in extrinsic stain and plaque removal |
|  | Aloe Barbadensis Leaf Juice | Vegetable-based ingredients | Emollient and antimicrobial agent; used to treat aphthous ulcers and to reduce the incidence of alveolar osteitis after third molar extraction surgeries; a mouth rinse containing A. vera was found to reduce gingival inflammation and gingival bleeding and was more effective than Listerine® in reducing the count of aerobic, microaerophilic and anaerobic bacteria |
|  | Aqua | Solvents | Solvent |
|  | Sodium Lauroyl Sarcosinate | Emulsifier | Acts as am emulsifier to improve the toothpaste foaming and thickening properties |
|  | Aroma (Natural Flavor) | Inactive - Fragrances | Fragrance additive |
|  | Xylitol | Whitening/plaque removal agents | Active in caries prevention + Effective against S. mutans |
|  | Escin | Antioxidant | Anti-edematous, anti-inflammatory, and venotonic properties |
|  | Cellulose gum | Thickening agents | Thickening agent |
|  | Melaleuca Alternifolia (Tea Tree) Leaf Oil | Vegetable-based ingredients | It is an essential oil extracted from the leaves of the Melaleuca alternifolia plant native to Australia. Antibacterial, anti-inflammatory, antiviral, and antifungal properties |
|  | Phenoxyethanol | Preserving agents | Preserving agent |
|  | Citric Acid | Whitening/plaque removal agents | Active in dental plaque removal and whitening teeth |
|  | Capsicum Annum Fruit Extract | Flavoring and Fragrance agents. Capsicum-derived ingredients are employed in cosmetics as skin-conditioning agents, external analgesics, flavoring agents, or fragrance components | Antioxidant, anti-inflammatory, antiseptic, and antimicrobial properties |
| **BIOEARTH DENT32 Junior Strawberry** | Sorbitol | Inactive - Sweeteners | Substitute for sugar used as a sweetening agent |
|  | Aqua (Water) | Solvents | Solvent |
|  | Cellulose gum | Thickening agents | Thickening agent |
|  | Glycerin | Wetting agents | Used to prevent toothpaste from drying out |
|  | Aroma (Flavor) | Inactive - Fragrances | Fragrance additive |
|  | Titanium Dioxide | Artificial - Colorant + Whitening/plaque removal agents | Active in extrinsic stain removal |
|  | Xylitol | Whitening/plaque removal agents | Active in caries prevention + Effective against S. mutans |
|  | Salvia Officinalis (Sage) Leaf Extract* | Vegetable-based ingredients | Anticancer and anti-mutagenic, anti-inflammatory and antinociceptive, antioxidant, antimicrobial, antifungal, antidementia, hypoglycemic, and hypolipidemic effects |
|  | Aloe Barbadensis Leaf Juice* | Vegetable-based ingredients | Emollient and antimicrobial agent; used to treat aphthous ulcers and to reduce the incidence of alveolar osteitis after third molar extraction surgeries; a mouth rinse containing A. vera was found to reduce gingival inflammation and gingival bleeding and was more effective than Listerine® in reducing the count of aerobic, microaerophilic and anaerobic bacteria |
|  | Benzyl Alcohol | Solvents | Solvent |
|  | Sodium Lauroyl Glutamate | Emulsifiers | Surfactant and foaming agent |
|  | Ethylhexylglycerin | Preserving agents | Preservative, antibacterial, surfactant, skin-conditioning, and emollient agent |
|  | Sodium Chloride | Abrasives | Abrasive agent |
| **Urtekram children’s toothpaste tutti-frutti** | **Calcium Carbonate** | Abrasives | Used as an abrasive, which helps remove extrinsic stains and plaque |
|  | Aqua | Solvents | Solvent |
|  | **Glycerin**** | Wetting agents | Used to prevent toothpaste from drying out |
|  | Aloe Barbadensis Leaf Extract* | Vegetable-based ingredients | Emollient and antimicrobial agent; used to treat aphthous ulcers and to reduce the incidence of alveolar osteitis after third molar extraction surgeries; a mouth rinse containing A. vera was found to reduce gingival inflammation and gingival bleeding and was more effective than Listerine® in reducing the count of aerobic, microaerophilic and anaerobic bacteria |
|  | Chamomilla Recutita Flower Extract* | Vegetable-based ingredients | It is an annual herb that grows in Southeast Europe and adjacent Asian countries, has been used for medicinal purposes since ancient times. The Egyptians considered it a sacred gift from the God 'Sun'. German chamomile is known as a "star" herb due to its aromatic and pharmacological properties, which include anti-inflammatory, antiulcerogenic, antimicrobial, antiseptic, antispasmodic, sedative, immunomodulatory, and wound-healing effects |
|  | **Xanthan Gum** | Thickening agents | Thickening and stabilizing agent |
|  | **Aroma** | Inactive - Fragrances | Fragrance additive |
|  | Commiphora Myrrha Oil | Vegetable-based ingredients | Myrrh is a resin extracted from the Commiphora myrrha tree native to northeastern Africa and southwest Asia. Its essential oil has a brownish-amber color with an earthy aroma and has been used for centuries in traditional Chinese medicine and Ayurvedic medicine. Has antibacterial, anti-inflammatory, anti-fungal, and antioxidant properties, making it useful for wound healing, pain management, and fighting infections and skin sores |
|  | Magnolia Officinalis Bark Extract | Vegetable-based ingredients | The houpo magnolia tree, native to China, has been used for centuries in traditional Chinese medicine. Anti-inflammatory, anticancer, antimicrobial, and antioxidant properties. Effective against *S. mutans* |
| **NeBioLina Bebé** | Sorbitol | Inactive - Sweeteners | Substitute for sugar used as a sweetening agent |
|  | Aloe Barbadensis Leaf Juice* | Vegetable-based ingredients | Emollient and antimicrobial agent; used to treat aphthous ulcers and to reduce the incidence of alveolar osteitis after third molar extraction surgeries; a mouth rinse containing A. vera was found to reduce gingival inflammation and gingival bleeding and was more effective than Listerine® in reducing the count of aerobic, microaerophilic and anaerobic bacteria |
|  | Hydrated Silica | Whitening/plaque removal agents | Used as an abrasive, active in extrinsic stain and plaque removal |
|  | Glycerin | Wetting agents | Used to prevent toothpaste from drying out |
|  | Xylitol | Whitening/plaque removal agents | Active in caries prevention + Effective against *S. mutans* |
|  | Avena Sativa (Oat) Kernel Extract* | Vegetable-based ingredients | Vegetable-based ingredient, abrasives, and bulking agents |
|  | Calcium Lactate | Wetting agents | Wetting agent, which helps with remineralization and prevents the formation of calculus |
|  | Aroma (Flavor)** | Inactive - Fragrances | Fragrance additive |
|  | Xanthan Gum | Thickening agents | Thickening and stabilizing agent |
| **Biricco Strawberry** | Aqua | Solvents | Solvent |
|  | Sorbitol | Inactive - Sweeteners | Substitute for sugar used as a sweetening agent |
|  | Hydrated Silica | Whitening/plaque removal agents | Used as an abrasive, active in extrinsic stain and plaque removal |
|  | Glycerin | Wetting agents | Used to prevent toothpaste from drying out |
|  | Erythritol | Inactive - Sweeteners | Sugar alcohol |
|  | Carboxymethylcellulose (cellulose gum) | Thickening agents | Thickening agent |
|  | Pyrus Cydonia Fruit Extract* | Vegetable-based ingredients | It’s an age-old fruit that originates from different parts of Asia and the Mediterranean and has been used as traditional folk medicine has used quince for many years, but scientific studies on its advantages are still in the preliminary stages. Current evidence suggests that it has antioxidant propertie |
|  | Chamomilla Recutita Flower Extract* | Vegetable-based ingredients | It is an annual herb that grows in Southeast Europe and adjacent Asian countries, has been used for medicinal purposes since ancient times. The Egyptians considered it a sacred gift from the God 'Sun'. German chamomile is known as a "star" herb due to its aromatic and pharmacological properties, which include anti-inflammatory, antiulcerogenic, antimicrobial, antiseptic, antispasmodic, sedative, immunomodulatory, and wound-healing effects |
|  | Malva Sylvestris Flower/Leaf Extract* | Vegetable-based ingredients | It’s a plant species native to Europe, northern Africa, and Asia. It promotes wound healing, and possessed antioxidant, anti-inflammatory, antibacterial, antiviral, and anticancer properties. It is also active against *S. mutans*, *S. aureus*, and *C. albicans* |
|  | Vaccinium Myrtillus Fruit Extract* | Vegetable-based ingredients | They are small blue berries that grow in Northern Europe and are similar in appearance to North American blueberries. They are an excellent natural source of anthocyanins, which provide several health benefits due to their antioxidant, anti-inflammatory, and antibacterial properties, particularly against *Staphylococcus aureus* |
|  | Aroma | Inactive - Fragrances | Fragrance additive |
|  | Glyceryl Caprate | Natural emulsifier and preserving agent | Natural emulsifier and preserving agent |
|  | Lauryl Glucoside | Surfactant | Foaming agent |
|  | Potassium Sorbate | Preserving agents | Preserving agent |
|  | Sodium Benzoate | Preserving agents | Preserving agent |
|  | Citric Acid | Whitening/plaque removal agents | Active in dental plaque removal and whitening teeth |
| **Radius Organic Coconut Banana Toothpaste for Kids** | **Glycerin**** | Wetting agents | Used to prevent toothpaste from drying out |
|  | Aloe Barbadensis Leaf Juice* (Aloe Vera) | Vegetable-based ingredients | Emollient and antimicrobial agent; used to treat aphthous ulcers and to reduce the incidence of alveolar osteitis after third molar extraction surgeries; a mouth rinse containing A. vera was found to reduce gingival inflammation and gingival bleeding and was more effective than Listerine® in reducing the count of aerobic, microaerophilic and anaerobic bacteria |
|  | Cocos Nucifera (Coconut) Fruit Powder* | Vegetable-based ingredients | Coconut fruit powder is derived from dried and ground coconut flesh. Studies have shown that it has various health benefits including hypocholesterolemic, anticancer, antihepatosteatotic, antidiabetic, antioxidant, anti-inflammatory, antimicrobial, and skin moisturizing properties. Effective in fighting against *S. mutans* and *C. albicans* |
|  | Erythritol* | Inactive - Sweeteners | Sugar alcohol |
|  | Water | Solvents | Solvent |
|  | Chamomilla Recutita (Chamomile) Flower Extract* | Vegetable-based ingredients | It is an annual herb that grows in Southeast Europe and adjacent Asian countries, has been used for medicinal purposes since ancient times. The Egyptians considered it a sacred gift from the God 'Sun'. German chamomile is known as a "star" herb due to its aromatic and pharmacological properties, which include anti-inflammatory, antiulcerogenic, antimicrobial, antiseptic, antispasmodic, sedative, immunomodulatory, and wound-healing effects |
|  | Organic Flavors* | Flavoring agents | Flavor |
|  | Cocos Nucifera (Coconut) Oil* | Vegetable-based ingredients | Antioxidant, anti-inflammatory, antimicrobial properties. Effective against *S. mutans* and *C. albicans*. |
|  | **Calcium Carbonate** | Abrasives | Used as an abrasive, which helps remove extrinsic stains and plaque |
|  | Cyamopsis | Emulsifiers | Natural thickener |
|  | Tetragonoloba (Guar) Gum* | Emulsifiers | Natural thickener |
|  | **Sodium Bicarbonate** | Whitening/plaque removal agents | Active in extrinsic stain and plaque removal |
|  | **Xanthan Gum** | Thickening agents | Thickening and stabilizing agent |
|  | Sodium Chloride | Abrasives | Abrasive agent |
|  | Quillaja Saponaria Wood Extract* | Foaming agent, cleansing agent, emulsifying, surfactant, and skin conditioning agent. | The extract has various uses such as foaming, cleansing, emulsifying, surfactant, and skin conditioning. It also has anti-inflammatory properties |
|  | **Citric Acid** | Whitening/plaque removal agents | Active in dental plaque removal and whitening teeth |
|  | **Tapioca Maltodextrin***** | Thickening agents | Thickening and bulking agent |
|  | Citrus Grandis (Grapefruit) Peel Oil* | Vegetable-based ingredients | It is a volatile oil that is derived from the peel of the grapefruit. It has potential antimicrobial, antitumor, antioxidant, anti-inflammatory, anticancer, antiepileptic, stomach tonic, cardiac stimulant, cytotoxic, hepatoprotective, nephroprotective, and anti-diabetic agent properties. Effective against *S. aureus* and *C. albicans* |
|  | **Eucalyptus Radiata (Eucalyptus) Leaf/Stem Oil*** | Vegetable-based ingredients | Eucalyptus leaves are a rich source of antioxidants, specifically flavonoids, such as catechins, isorhamnetin, luteolin, kaempferol, phloretin, and quercetin. These flavonoids possess anti-inflammatory properties, which help reduce pain, and exhibit antibacterial activity |
|  | **Melaleuca Alternifolia (Tea Tree) Leaf Oil*** | Vegetable-based ingredients | It is an essential oil extracted from the leaves of the Melaleuca alternifolia plant native to Australia. Antibacterial, anti-inflammatory, antiviral, and antifungal properties |
|  | Rosmarinus Officinalis (Rosemary) Leaf Oil* | Vegetable-based ingredients | It is an evergreen shrub with needle-shaped leaves and a woody scent. Numerous researchers are currently investigating its potential health advantages. The plant has been found to possess analgesic effects and anti-inflammatory properties. It has also been found to be effective against *S. mutans* and *C. albicans* |
| **Lavera Naturkosmetik Kids** | Sorbitol | Inactive - Sweeteners | Substitute for sugar used as a sweetening agent |
|  | Aqua | Solvents | Solvent |
|  | Xylitol | Whitening/plaque removal agents | Active in caries prevention + Effective against S. mutans |
|  | Calcium Carbonate | Abrasives | Used as an abrasive, which helps remove extrinsic stains and plaque |
|  | Glycerin | Wetting agents | Used to prevent toothpaste from drying out |
|  | Xanthan Gum | Thickening agents | Thickening and stabilizing agent |
|  | Aloe Barbadensis Leaf Juice | Vegetable-based ingredients | Emollient and antimicrobial agent; used to treat aphthous ulcers and to reduce the incidence of alveolar osteitis after third molar extraction surgeries; a mouth rinse containing A. vera was found to reduce gingival inflammation and gingival bleeding and was more effective than Listerine® in reducing the count of aerobic, microaerophilic and anaerobic bacteria |
|  | Fragaria Vesca Fruit Extract** | Vegetable-based ingredients | Contains various biologically active phenolic compounds like tannins, anthocyanins, flavonoids, and phenolic acids. These compounds contribute to its antioxidant, antimicrobial, anti-inflammatory, anticoagulant, and vasodilatory effects |
|  | Calendula Officinalis Flower Extract** | Vegetable-based ingredients | Calendula extract is an extract from fresh calendula flower heads that contains mono and sesquiterpenes, triterpenes, phenol acids, flavonoids, tannins, and carotenoids. It has been found to prevent gingivitis, periodontal disease, stomatitis, and halitosis, and it is effective in wound healing |
|  | Glycyrrhiza Glab Root Extract** | Vegetable-based ingredients. Vegetable-based ingredients. It’s a sweet and soothing herb originating from the Glycyrrhiza glabra plant, has been widely used as a sweetener and flavoring agent in food and medicine production | Possesses anti-inflammatory, anti-oxidant, and anti-microbial properties, effective in treating dental caries, gingivitis, periodontitis, aphthous ulcers, and oral cancer. Reduces the count of *S. mutans* |
|  | Sodium Lactate | Humectant | **Humectant, moisturizer, and preservative** |
|  | Aroma*** | Inactive - Fragrances | Fragrance additive |
|  | Limonene*** | Inactive - Fragrances | Flavor and fragrance additive |
| **Jack n’ Jill Flavor Free** | Xylitol | Whitening/plaque removal agents | Active in caries prevention + Effective against S. mutans |
|  | Purified Water | Solvents | Solvent |
|  | **Glycerin (coconut derived)** | Wetting agents | Used to prevent toothpaste from drying out |
|  | Silica | Abrasives | Abrasive agent |
|  | **Xanthan Gum** | Thickening agents | Thickening and stabilizing agent |
|  | **Potassium Sorbate (naturally derived)** | Preserving agents | Preserving agent |
|  | **Citric Acid** | Whitening/plaque removal agents | Active in dental plaque removal and whitening teeth |
| **G.U.M Kids 3+** | Aqua | Solvents | Solvent |
|  | **Sorbitol** | Inactive - Sweeteners | Substitute for sugar used as a sweetening agent |
|  | **Hydrated Silica** | Whitening/plaque removal agents | Used as an abrasive, active in extrinsic stain and plaque removal |
|  | Isomalt | Inactive - Sweeteners | Sugar alcohol |
|  | **Cellulose gum** | Thickening agents | Thickening agent |
|  | **Aroma** | Inactive - Fragrances | Fragrance additive |
|  | **Disodium Phosphate** | Preservative and a stabilizer | Stabilises dental products and controls acidity |
|  | **Lauryl Glucoside** | Surfactant | Foaming agent |
|  | **Sodium Saccharin** | Inactive - Sweeteners | Artificial sweetener |
|  | **Sodium Benzoate** | Preserving agents | Preserving agent |
|  | **Sodium Fluoride 500 ppm** | Active ingredient | Anti-caries activity, whitening effects, halitosis control |
|  | **Sodium Phosphate** | Emulsifier | Emulsifier |
|  | **Cl 73360** | Inactive - Artificial colorant | Colorant |
| **THE HUMBLE CO. kids Strawberry toothpaste** | **Sorbitol** | Inactive - Sweeteners | Substitute for sugar used as a sweetening agent |
|  | Aqua | Solvents | Solvent |
|  | **Hydrated Silica** | Whitening/plaque removal agents | Used as an abrasive, active in extrinsic stain and plaque removal |
|  | **Xylitol** | Whitening/plaque removal agents | Active in caries prevention + Effective against S. mutans |
|  | **Xanthan Gum** | Thickening agents | Thickening and stabilizing agent |
|  | **Lauryl Glucoside** | Surfactant | Foaming agent |
|  | **CI 7789** | Artificial - Colorant + Whitening/plaque removal agents | Active in extrinsic stain removal |
|  | **Sodium Fluoride 1000 ppm** | Active ingredient | Anti-caries activity, whitening effects, halitosis control |
|  | Aloe Barbadensis Leaf Juice | Vegetable-based ingredients | Emollient and antimicrobial agent; used to treat aphthous ulcers and to reduce the incidence of alveolar osteitis after third molar extraction surgeries; a mouth rinse containing A. vera was found to reduce gingival inflammation and gingival bleeding and was more effective than Listerine® in reducing the count of aerobic, microaerophilic and anaerobic bacteria |
|  | **Aroma** | Inactive - Fragrances | Fragrance additive |
|  | Rebaudioside A | Inactive - Artificial sweetener | A highly purified form of stevia. Bio-sweetener. Anti-hypertensive, anti-obesity, anti-diabetic, antioxidant, anti-cancer, anti-inflammatory, and antimicrobial effects and improvement of kidney function |
|  | **Sodium Benzoate** | Preserving agents | Preserving agent |
|  | Potassium Sorbate | Preserving agents | Preserving agent |
|  | **Citric Acid** | Whitening/plaque removal agents | Active in dental plaque removal and whitening teeth |
|  | **Pentylene Glycol** | Humectant | A preservative, solvent, and humectant |
|  | Glyceryl Caprylate | Emulsifier | Emulsifier |
| **NUK Tooth and Gum Cleanser for Babies 3-12 months** | Aqua | Solvents | Solvent |
|  | Glycerin | Wetting agents | Used to prevent toothpaste from drying out |
|  | Sorbitol | Inactive - Sweeteners | Substitute for sugar used as a sweetening agent |
|  | Propylene Glycol | Surfactant | Act as penetration enhancers |
|  | Pectin | Thickening agents | thick |
|  | Xanthan Gum | Thickening agents | Thickening and stabilizing agent |
|  | Hydrated Silica | Whitening/plaque removal agents | Used as an abrasive, active in extrinsic stain and plaque removal |
|  | Aroma | Inactive - Fragrances | Fragrance additive |
|  | Calcium Lactate | Wetting agents | Wetting agent, which helps with remineralization and prevents the formation of calculus |
|  | Lactoferrin | Protein | Has been found to possess anti-inflammatory, anti-microbial, and anti-viral properties. Effective against *S. mutans* |
|  | Lysozyme | Enzyme | Antimicrobial. Effective against *S. mutans* |
|  | Lactoperoxidase | Enzyme | Has anticariogenic, antibacterial, antifungal, and antiviral properties. Effective against *S. mutans* |
